# Nanoparticles and Nanomaterials: A Review from the Standpoint of Pharmacy and Medicine

**DOI:** 10.3390/pharmaceutics17050655

**Published:** 2025-05-16

**Authors:** Gleb V. Petrov, Alena M. Koldina, Oleg V. Ledenev, Vladimir N. Tumasov, Aleksandr A. Nazarov, Anton V. Syroeshkin

**Affiliations:** 1 Department of Pharmaceutical and Toxicological Chemistry, Medical Institute, RUDN University, 6 Miklukho-Maklaya Street, 117198 Moscow, Russia; koldina_am@pfur.ru (A.M.K.); syroeshkin_av@pfur.ru (A.V.S.); 2 Department of Biology, Lomonosov Moscow State University, Leninskie Gory, 119234 Moscow, Russia; ya@olegledenev.ru; 3 Department of Pharmaceutical Chemistry and Organization of Pharmaceutical Business, Faculty of Medicine, Lomonosov Moscow State University, GSP-1, Leninskie Gory, 119991 Moscow, Russia; vntumasov@yandex.ru

**Keywords:** nanoparticles, nanomaterials, pharmacy, cancer therapy, drug delivery, pharmaceutical chemistry, theranostics, target delivery, quality control

## Abstract

Nanoparticles (NPs) represent a unique class of structures in the modern world. In comparison to macro- and microparticles, NPs exhibit advantages due to their physicochemical properties. This has resulted in their extensive application not only in technical and engineering sciences, but also in pharmacy and medicine. A recent analysis of the scientific literature revealed that the number of articles related to the search term “nanoparticle drugs” has exceeded 65,000 in the last decade alone, according to PubMed. The growth of scientific publications on NPs and nanomaterials (NMs) in pharmacy demonstrates the rapidly developing interest of scientists in exploring alternative ways to deliver drugs, thereby improving their pharmacokinetic and pharmacodynamic properties, and the increased biocompatibility of many nanopharmaceuticals is a unique key to two mandatory pharmaceutical requirements—drug efficacy and safety. A comprehensive review of the literature indicates that the modern pharmaceutical industry is increasingly employing nanostructures. The exploration of their physicochemical properties with a subsequent modern approach to quality control remains the main task of modern pharmaceutical chemistry. The primary objective of this review is to provide a comprehensive overview of data on NPs, their physicochemical properties, and modern approaches to their synthesis, modification of their surface, and application in pharmacy.

## 1. Introduction

The first documented example in the history of nanoscale was in 1905, when the theoretical physicist Albert Einstein described it in his doctoral dissertation. Einstein applied a mathematical model describing the movement of molecules in water to an aqueous solution of sugar and demonstrated that the size of the molecules is smaller than the micro range [1]. The study of the nanoworld has developed since the beginning of the 20th century within the framework of colloidal chemistry [2]. In 1959, at the American Physical Society, Richard P. Feynman presented his lecture “There is plenty of room at the bottom” [3], thereby laying the foundations for the development of the scientific discipline of nanotechnology. After Feynman’s lecture, the scientific world was excited by the idea of studying “invisible” particles. Consequently, instruments for visualizing NPs, such as scanning probe and atomic force microscopes [4,5,6] and dynamic light scattering [7], underwent active development. The scientific discoveries referenced above have resulted in the almost daily use of NPs in a variety of industries, including technology, engineering, pharmacy, and medicine.

One of the first reviews, titled “Nanoparticles and nanocapsules—new dosage forms in the nanometer size range”, was published in 1978 [8]. In the period spanning from that moment until approximately the beginning of the 2000s, only a few reviews were published, and only since 2004 has been an increase in the interest in NPs in the field of scientific publications, with a corresponding increase in the number of reviews (more than 10-fold). Consequently, over the past two decades, literature has emerged concerning a specific type of NPs. The initial publications were reviews of metal nanostructures [9,10]. However, these particles have been investigated either from the point of view of their physicochemical properties and toxicity or from the point of view of their application in medicine [11,12,13]. A substantial number of analogous reviews have already been published, encompassing not only metal NPs, but also other types of ultrasmall particles. Many of these reviews are accompanied by meticulous tables that provide detailed descriptions of the synthesis, properties, and applications of NPs [14]. However, the existing literature on the application of NPs in medicine and pharmacy is largely limited in scope and focused on specific research vectors. These reviews are of particular importance to modern scientific society, as they provide detailed information on specific cases. The majority of review papers in the field of pharmacy are focused on two main areas. Firstly, there is the study of specific types of nanopharmaceuticals that have already been registered. Secondly, the focus is on the various modifications of NPs for the delivery of specific active substances.

The present review does not claim to supersede extant works but rather aims to provide a comprehensive summary of the current state of information concerning nanostructures from the perspective of pharmaceutical and medical sciences. Therefore, the purpose of this work is to summarize the existing knowledge on different types of NPs, their physicochemical properties, synthesis methods, modification of their surface, applications in pharmacy and medicine and, of course, quality control as the main task of pharmaceutical chemistry.

## 2. Classification of NMs

**Figure 1:**
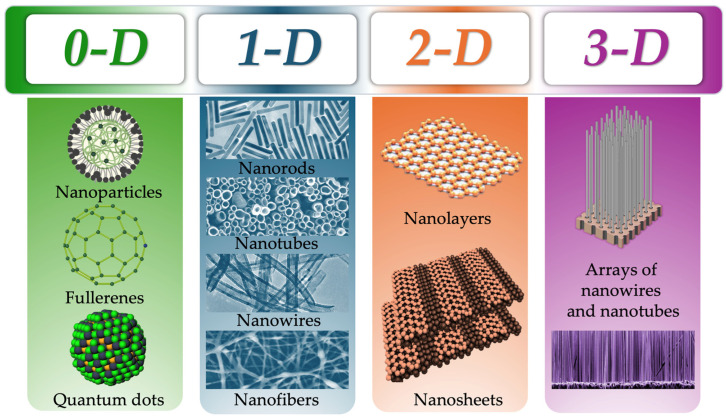
The classification of NMs by their dimensions, where 0-D NMs have all three dimensions in the nanoscale, they include NPs and quantum dots; 1-D NMs have two of the three dimensions in the nanoscale, including nanorods, nanotubes, nanowires and nanofibers; 2-D NMs have at least one dimension in nanoscale, representatives are nanolayers and nanosheets; 3-D NMs none of the three dimensions are included in the nanoscale, they include arrays of nanowires and nanotubes [18].

It has been established that NPs are defined as particles of the dispersed phase with sizes ranging from 1 to 100 nm [15]. This small scale declare unique physical and chemical properties of this objects and they become subjects of the quantum laws [16]. It is important to note that the term NMs is frequently mentioned in the scientific literature. It should be noted that NMs are not synonymous with NPs but rather represents a distinct concept comprising a subset of materials derived from a specific number of NPs. More accurate definition of NMs is as follows: these are materials in which at least one of the dimensions is on the nanoscale [17]. According to the aforementioned definition, it is common for NMs to be classified as follows (Figure 1).

NMs classified into four dimensions (Figure 1), from zero (0-D), where all three dimensions are in the nanoscale, to three-dimensional (3-D), where none of the dimensions are in the nanoscale [18,19]. In the fields of pharmacy and medicine, 0-D and 1-D NMs are the most used due to their ultra-small dimensional characteristics and penetrating properties [20]. The zero-dimensional materials encompass fullerenes, NPs, and quantum dots. The most common applications of these materials, as outlined in the relevant publications, are as follows: drug delivery [21,22,23,24], use as contrast agents due to their fluorescent properties [25,26], and cancer therapy [27,28,29]. In contemporary immunology, 0-D NMs is frequently applied, developing virus-like NPs known as virus like particles (VLPs) [30]. These immunobiological pharmaceuticals are the subject of ongoing research and development, with the potential for production of novel recombinant vaccines that could result in the complete absence of side effects during vaccination.

In the context of the 1-D class of the NMs representatives, the focus of the authors is predominantly directed towards nanotubes [19,31]. The first documented reference to carbon nanotubes in the scientific literature was in 1992, when the Japanese physicist Sumio Iijima and his team described the process of growing carbon nanotubes using electron microscopy [32]. From a medical standpoint, the most significant surge in interest for this subject commenced in 2019. Most articles published on the subject of nanotubes are related to biomedicine [33], medicine [34,35] and cytology [36]. The application of nanotubes in pharmacy is growing in popularity, with the possibility of using them for targeted drug delivery being explored [37,38,39,40]. As outlined in the following article, the immunostimulating properties of carbon nanotubes are described [41]. The authors of the article highlight the main advantages of nanotubes as adjuvants for vaccines, where, referring to their physicochemical properties, they propose to include them in modern vaccines to increase the effectiveness of immunization [42,43].

## 3. Classification of NPs

**Figure 2:**
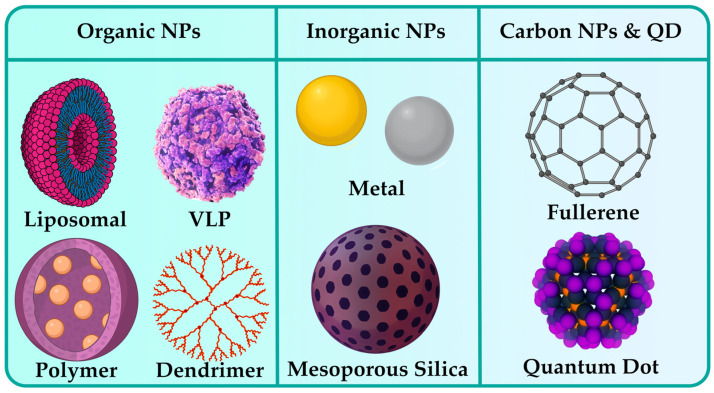
The classification of NPs, where liposomal, VLP, polymer, and dendrimer particles are classified as an organic; metal and mesoporous silica NPs as inorganic; and carbon-based particles and quantum dots (QD) as separate classes.

Many authors classify NPs primarily according to the nature of their origin, dividing them into particles of an organic nature and particles of an inorganic nature, and carbon NPs are also classified in a separate class [44]. In turn, organic particles include liposomal, virus-like particles (VLP), polymeric, and dendrimeric. Inorganic NPs are represented by two types: metallic and mesoporous silica particles. Carbon nanostructures, including fullerenes and quantum dots, are posited to be evaluated independently. Figure 2 provides a visual representation of the classification of NPs according to their natural origin. More detailed analysis of each class is presented in the subsequent sections.

### 3.1. Organic NPs

Representatives of organic NPs are those particles in which carbohydrates and various protein structures can be found [44]. As the focus of this review is aimed on the pharmaceutical industry, it is worthwhile to mention those NPs that are widely used in this area. Liposomal, protein, and polymeric NPs are actively used both in the targeted delivery of drugs [45,46,47] and in the development of modern immunobiological pharmaceuticals, as well as in a variety of therapies.

#### 3.1.1. Liposomal NPs

In 1961, during experimentation with an electron microscope for observation of dry phospholipids in Cambridge, British hematologist Alec Bangham made a remarkable discovery. He observed an unidentified structure that would later be named “liposome”, deriving its name from lysosomes, which were also studied in the laboratory where Bangham was working at the time. In 1964, the results were published in the Journal of Molecular Biology [48]. Subsequently, the application of liposomes as drug delivery systems was proposed. In the review conducted by Jeong M. [49], the potential application of liposomal NPs (LNPs) as effective RNA delivery systems was explored. Due to their physicochemical properties, LNPs are able to penetrate various barriers inside the human organism [45,50,51]. Therefore, drugs based on LNPs can prolong the effect of the active substance [52,53], modify pharmacokinetic properties, improve pharmacological properties, and increase bioavailability [54]. From an immunological perspective, the delivery of drug substances comprising RNA poses a significant challenge, the resolution of which would facilitate the development of novel vaccine formulations [51]. It is evident that the potential of LNPs extend beyond the aforementioned application; a significant pool of articles focuses on the potential of these particles in cancer therapies [55,56]. It has been posited by numerous authors that liposomal particles have the potential to function as delivery agents in the context of chemotherapy [57,58,59].

#### 3.1.2. VLPs as Contemporary Immunobiological Nanostructures

The aforementioned VLPs can be classified as protein NPs. These particles have been the focus of research since the late 1960s, when VLPs of a particular type derived from Hepatitis B virus were first described in 1968 [60]. A number of vaccines based on the VLP are currently available on the pharmaceutical market. The first was a Hepatitis B vaccine, which was approved by the US Food and Drug Administration (FDA) in 1986 [61]. Following this development, vaccines against human papillomavirus [62,63], human immunodeficiency virus [64,65], Ebola [66,67], dengue fever [68,69], rotavirus [70], SARS-CoV-2 [30], etc., officially entered the market. It has been established that a number of these vaccines are currently commercially licensed and used for the active prevention of viral infections, most of the aforementioned vaccines undergoing clinical trials at the final stage.

#### 3.1.3. Polymeric and Dendrimeric NPs

**Figure 3:**
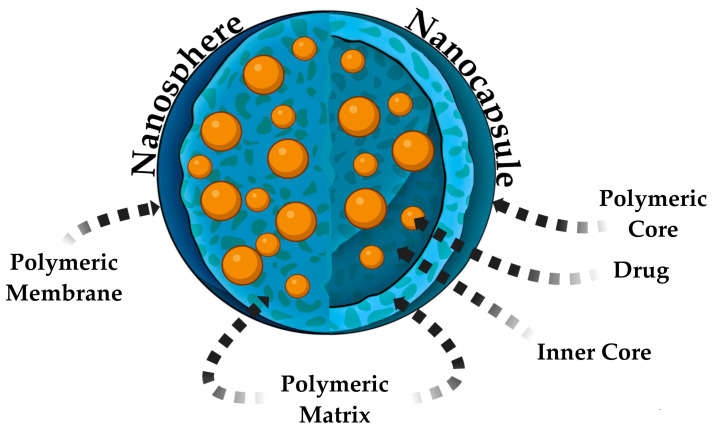
Comparison of the structure of nanospheres and nanocapsules. Nanospheres have the capacity to contain APIs within the polymeric matrix, whereas nanocapsules contain APIs within the polymeric core.

In this review, the focus will now shift to the final group of organic nature NPs—polymer and dendrimer particles. Polymeric NPs are a novel pharmaceutical dosage form in the field of pharmacy. The earliest references to polymeric NPs in scientific publications date back to the late 1970s [8], and subsequent articles have actively developed applications for encapsulating pharmaceuticals. These particles have a high intracellular uptake and biocompatibility with cells and tissues due to their size, which bring them closer to the size of protein molecules in the human organism [71], and their mechanical properties such as flexibility [72]. Polymeric NaPs can be classified into two primary categories: nanocapsules [73,74] and nanospheres [75,76] (Figure 3). Before discussing the differences between them, it is noteworthy that the synthesis of polymer NPs can be based on the process of obtaining an oil-in-water emulsion, that is, a system comprising two phases, wherein one phase is aqueous, encompassing water and surfactants, and the second phase is organic, encompassing polymeric compounds, oil and an active pharmaceutical ingredient (API) [77]. In addition, chemical methodologies exist for the synthesis of hybrid polymer nanocapsules that do not necessitate the production of an emulsion. Rather, radical polymerization reactions are employed, utilizing a variety of monomers [78].

Polymer nanospheres are defined as particles consisting of a polymer membrane and a matrix, within which a pharmaceutical substance is encapsulated (Figure 3). In contrast, nanocapsules exhibit a more intricate structural composition, encompassing, in addition to the polymer matrix, a core, and an inner core (Figure 3). Both variations of polymer particles are used in pharmacy. The main difference is that nanospheres can only encapsulate drugs and release them exponentially, whereas nanocapsules increase the potential to modify the pharmacokinetic properties of drugs due to their complex structures [79]. All the above-mentioned morphological characteristics make it possible to use these particles in a completely different range of tasks.

One of the varieties of polymer particles is considered to be dendrimers, which are NMs based on highly branched polymers with a central core and several branches that end in functional groups [47]. Their research, grounded in data from scientific publications, was initiated during the early 1990s. Dendrimers are usually applied as linker molecules in tumor diagnostics [80]. However, their synthesis was described previously in the scientific literature [81]. At this stage of the research, the possibility of their application as drug carriers is being actively investigated [47].

### 3.2. Inorganic NPs

Inorganic particles are defined as samples that are devoid of carbon [82]. This may encompass the pervasive noble metal NPs, which are applicated in anticancer therapy, as adjuvants in radiation therapy [83], and as delivery vehicles for pharmaceuticals [84]. The most common representatives of this class are gold NPs. The history of scientific research in the field of gold particles dating back to the 1850s, when the English scientist Michael Faraday accidentally prepared a ruby solution of colloidal gold with unique optical properties [85]. Faraday had previously posited that the color of the solution is dependent on the size of the gold particles. The study of the light scattering properties of suspended gold particles has enabled the interpretation of the Faraday–Tyndall effect.

AgNPs are equally popular [86]. Their application in the fields of medicine and pharmacy is well known. A wide range of coatings incorporating these NPs are well established, and they are actively incorporated into a variety of bandage materials [87]. In the field of pharmacy, AgNPs are applicated as antiseptic agents in both liquid and soft dosage forms. A distinctly promising application of AgNPs in pharmaceuticals is their combination with antibiotics. A vast array of publications is dedicated to investigating the synergistic interplay between AgNPs and a variety of antibiotics [88,89,90], which exhibit a positive trend in their therapeutic impact on pathogenic microorganisms such as *Staphylococcus aureus*, *Escherichia coli*, and *Pseudomonas aeruginosa* [91,92,93]. This synergy enables the enhancement of bacterial organisms’ susceptibility to antibacterial agents, which consequently results in a reduction of the prescribed dosage and an attenuation of bacterial resistance towards specific antibiotics [94].

In addition to AuNPs and AgNPs, particles of metal oxides (TiO_2_, ZnO, Fe_2_O_3_, etc.) have been demonstrated to be employed as platforms for the delivery of drugs and for the diagnosis of diseases [95,96,97]. Several publications delve into the application of superparamagnetic NPs of iron oxide in the context of drug delivery systems, diagnostic instruments for magnetic resonance imaging, and exhibiting theranostic potential in the field of oncology [98,99]. The authors of the next scientific publications highlight the successful application of nanocomposites based on TiO_2_ as “smart” systems for the targeted delivery of anticancer pharmaceuticals. For example, one article presents research into pH-responsive drug delivery systems for daunorubicin based on TiO_2_ NPs as a carrier [100]. The authors observed a marked enhancement of the antitumor efficacy of the active agent in conjunction with these particles. Yin et al. [101] emphasize the therapeutic efficacy of mesoporous TiO_2_ particles, which exhibit promise in both photodynamic therapy and pharmaceutical delivery. In addition to the aforementioned particles, ZnO NPs also find their application in the treatment and detection of oncological disease [102]. Nonetheless, it has also been outlined that they can be employed as a potential antimicrobial drug delivery mechanism. The antibacterial efficacy of ciprofloxacin combined with ZnO NPs was demonstrated [103]. This combination enabled to effectively inhibit the growth of both *Staphylococcus aureus* and *Pseudomonas aeruginosa*, thereby achieving a significant reduction in their proliferation [103].

Silicon particles may also be categorized as representatives of the class of inorganic NPs. Frequently, scientific works dedicated to the exploration of silicon particles as carriers for drug delivery describe their mesoporous configurations [104,105]. They exhibit distinctive physicochemical characteristics that render them suitable for application in the treatment of a wide range of medical conditions. The scientific literature describes their application as agents for targeted delivery in cardiovascular [106], oncological [107,108], ophthalmological, and bacterial diseases [109].

### 3.3. Carbon-Based NPs

Another class of NPs distinguished by their nature is carbon particles. These representatives consist entirely of carbon atoms. It is pertinent to discuss fullerenes in this context. The study of fullerenes was a subject of intense research in the late 1980s and early 1990s. A seminal contribution in this field was the paper published by Friedman in 1993 in the Journal of the American Chemical Society, which explored the possibility of inhibiting the activity of HIV-1 protease by fullerenes [110]. From the perspective of contemporary immunology, this scientific endeavor can be described as groundbreaking, as the approach outlined within its pages provides a wealth of intellectual stimulation for the scientific community. For example, it has been demonstrated that fullerenes can be employed as antiviral agents during the SARS-CoV-2 pandemic of 2019 [111]. The authors of the paper investigate the application of carbon NPs, which are presented as an effective tool against the virus. Thus, the potential of applicating carbon particles as biosensors for detecting SARS-CoV-2 and their ability to inhibit viral replication are being explored [112]. As with previous types of NPs, there is the possibility of employing carbon nanostructures for the delivery of drugs in the form of metal-encapsulated complexes [113].

### 3.4. Quantum Dots

Quantum dots are extensively explored as precise drug delivery systems, agents for cell visualization, and instruments for theranostic purposes [114], serving as a means of both diagnosis and therapy with a single medication. The most intense interest in these particles emerged following the 2023 Nobel Prize in Chemistry, which was awarded to three scientists for their research on quantum dots, encompassing both their detection and synthesis. Although the history of their development goes back to the early 1980s, when the first quantum dots were synthesized [115,116]. In the year 1993, researchers from the Massachusetts Institute of Technology put forward a methodology for the production of quantum dots with precisely controlled dimensions [117]. These groundbreaking discoveries have enabled to re-examine the potential of nanotechnology. Despite the fact that the use of quantum dots gained widespread popularity only two years ago, research on the topic of quantum dots in the areas of medicine and pharmacy started in the early 2000s. The initial medical application of quantum dots involved their use in bio-imaging in the field of oncology [118]. In contemporary scientific circles, there is a vigorous exploration of the potential applications of quantum dots in the realm of medical treatment [119], as well as their utilization as genetically engineered biosensors [120]. Within the realm of pharmaceutical science, like with other forms of nanostructures, there is an ongoing exploration of the potential for drug delivery [121].

A more comprehensive description and application of NPs of various nature of origin is provided in Section 7 “Contemporary Application of NPs”.

## 4. Physicochemical Properties of NPs

After the analyzation of the classification of NMs and NPs, it is worthwhile to examine their distinctive physicochemical properties. Due to their ultra-small size, in addition to the fundamental physical laws, quantum laws also come into force. Here, we delve into each characteristic with a more detailed view.

### 4.1. Morphological Properties of NPs

Morphology is a comprehensive description of the morphological features of nanoscale objects, encompassing their dimensions, shapes, and aggregate structure. The size of NPs in pharmacy is a fundamental criterion, depending on the size characteristics, their pharmacokinetic and pharmacodynamic parameters will change. As was previously stated, NPs vary in size from 1 to 100 nm. This exact range ensures higher intracellular absorption. Particles with a size smaller than 10 nm can easily pass through the walls of blood vessels, overcoming tissue barriers, and are subsequently excreted by the kidneys [122]. By varying the classes of NPs during the creation of transport systems for drug delivery, the property of passing through various organism barriers, including the blood–brain barrier, plasma membranes, and capillary walls, can also be improved [123,124].

**Figure 4:**
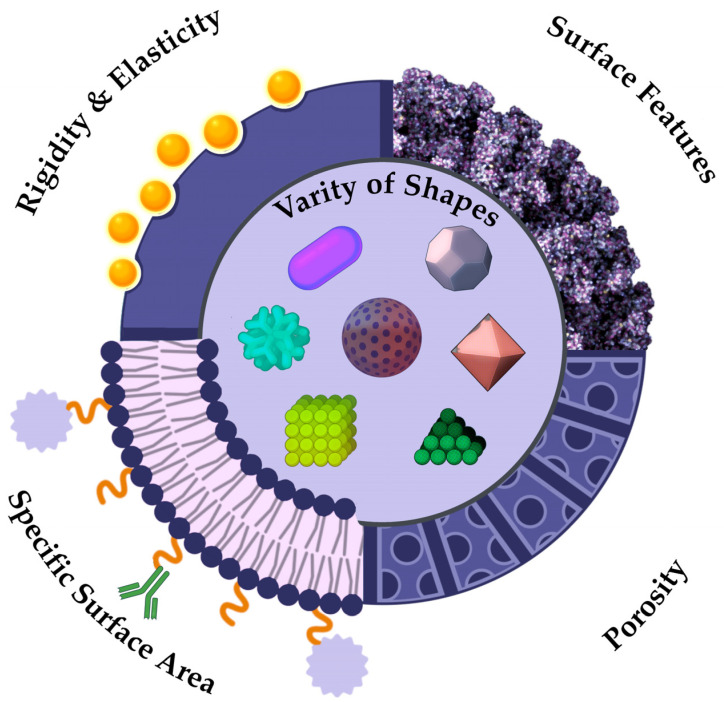
Morphological, topographical, and mechanical characteristics of NPs. From the perspective of morphological and topographical features, the figure exhibits a variety of NPs form factors, their external surfaces, and internal configurations (Varity of Shapes; Surface Features; Porosity). Mechanical properties are demonstrated by the example of the rigidity and elasticity of NPs. Specific surface area of NPs is also presented, which plays an important role in the investigation of their capacity for effective drug delivery.

In the contemporary world, NPs assume a diverse array of shapes, rendering it possible to introduce a novel classification based on formfactor, and each passing year it will only expand (Figure 4). At this juncture, it is possible to distinguish between standard spherical and irregularly shaped particles. If the concept of spherical particles is more or less self-explanatory—these are particles that closely resemble ideal spheres and are most commonly represents of metal NPs such as gold, silver, or zinc, as well as quantum dots, carbon particles, latex spheres and various protein structures like bovine serum albumin—then the realm of non-spherical particles presents a multifaceted landscape of diverse particle forms [125]. To illustrate an example of non-spherical NPs, let us consider the VLP structure. Due to the presence of viral protein domains on their surfaces, they assume a conformation that is not ideally spherical [126]. Nanotubes, nanofibers, peptide modified NPs, polymers, and other nanostructures that deviate from a spherical shape or feature surface inclusions are collectively referred as non-spherical structures (Figure 4). The form serves only as an indirect characteristic, but this assumption is inaccurate. The study presents a comparative analysis of spherical and rod-like particles in the context of pharmaceutical delivery systems [127]. The team of scientists underscores the benefits of spherical particles, as they are more readily assimilated by the body. The influence of a large ratio of surface area to volume of anisotropic structures will determine increased efficiency in combining NPs with biologically active objects. The shape of particles influences not only their pharmacological and pharmacokinetic properties, but also their patterns of accumulation within the body. Thus, the scientific research indicates that regular spherical particles tend to accumulate predominantly in liver cells, irregular spherical particles in the spleen, and discoid particles in the heart [128].

### 4.2. Mechanical Properties of NPs

The distinctive feature of NPs is their mechanical properties, which diverge from those of micron-sized particles. Mechanical properties are a characteristic describing the ability of materials to resist the action of environmental factors, determining exactly how the sample reacts to various loads or external influences [129]. The following mechanical characteristics are interpreted for nanoscale particles: brittleness, strength, hardness, plasticity, elasticity (Figure 4) [130]. The durability of metallic and organic substances is superior to that of inorganic and non-metallic compounds, which are often more brittle. For example, micro-sized particles of Fe_2_O_3_, SiO_2_, and Al_2_O_3_ will have increased brittleness, while their nano-analogues show improved strength and elasticity characteristics [131,132,133]. In the field of medicine, nanocomposite materials are being actively explored and implemented in dental practice. Thus, inorganic NPs are employed to enhance the mechanical properties of dental materials [134]. Mechanical properties also play an important role in the field of drug delivery. The pharmacodynamic properties of NPs are susceptible to variation, depending on their stiffness. Particles with a low degree of rigidity are known to elicit a reduced response from macrophages, a factor which contributes to a prolonged circulation time in the bloodstream [135]. More rigid particles, which exert significant pressure on cell membranes, is known to induce endocytosis [136]. The study of the mechanical properties of NPs is necessary to produce modern pharmaceuticals with targeted delivery. However, the properties described above are closely related to other physicochemical characteristics, including specific surface area and interaction forces.

### 4.3. Specific Surface Area

One of the most significant physical characteristics that influence the reactivity and adsorption properties of nanostructures is their specific surface area (SSA) (Figure 4). According to the IUPAC, the definition of SSA is interpreted as a property of solids, which is determined by the total cumulative surface area of a material per unit of mass (m^2^/kg) or volume (m^2^/m^3^) [137]. As stated in the recommendation of the European Commission: “Nanomaterials means a natural, random or artificial material containing nanoparticles in an unbound state, in the form of an aggregate or agglomerate, in which for 50% or more of the particles in a quantitative size distribution, one or more external dimensions range from 1 nm to 100 nm” [138]. Alternatively, it is indicated that the material should be considered as falling under this definition if the SSA of the object by volume exceeds 60 m^2^/cm^3^ [139]. The value of the surface area of the NPs may be indicative of their effectiveness in transporting drugs, since the higher the SSA, the greater the amount of the drug can be adsorbed on the surface of the particle (Figure 4) [140].

### 4.4. Types of Intermolecular Interactions

The forces of interaction between nanostructures represent a significant physical characteristic. They are a fundamental link in the uniqueness of the physicochemical properties of NPs.

#### 4.4.1. Van der Waals Forces

In the context of intermolecular forces of interaction between NPs, the van der Waals forces merit consideration as a point of departure. These are weak intermolecular interaction forces that arise due to temporary dipole moments formed by particles, acting at short distances up to 1 nm. There are three types of these forces—orientation, induction, and dispersion.

**Figure 5:**
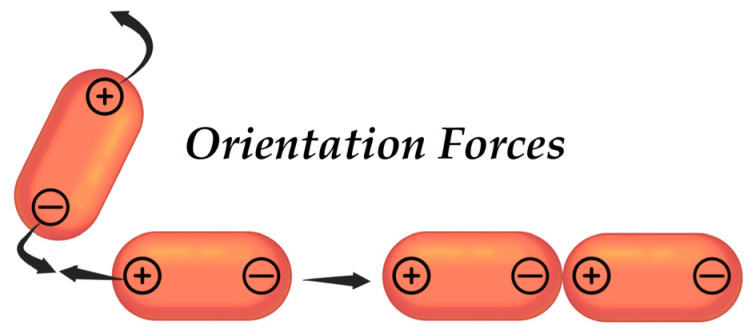
Visualization of the orientation forces of interaction.

Orientation forces arise between polar particles that possess permanent dipoles (Figure 5). When two particles come into proximity, they may realign their charges in accordance with the principle of “positive charge attracts negative charge” and vice versa, which leads to the emergence of attractive forces [141]. The Keesom’s energy of attraction of particles can be described by the following Equation (1):

(1) $$E_{K}=\frac{-2\mu_{1}\mu_{2}}{4\pi \varepsilon_{0}r^{3}}$$

where $\mu_{1}$ and $\mu_{2}$ are the dipole moments of the interacting dipoles, *r* is the distance between them, and $\varepsilon_{0}$ is the electric constant.

The Keesom forces are more pronounced when particles are in proximity to each other, and as they move away from one another, their intensity diminishes. Additionally, when the temperature of the sample is increased, both the orientation of the particles and their speed of movement are subject to alteration, which may also affect the influence of the orientation forces of interaction.

**Figure 6:**
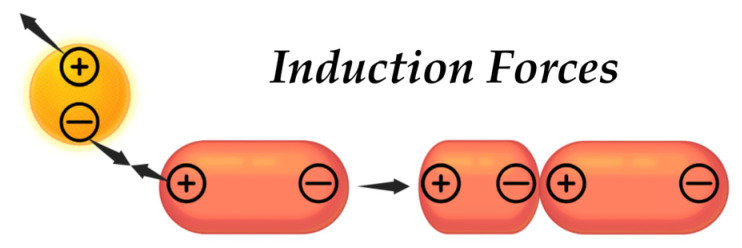
Visualization of the induction forces of interaction.

The induction interaction refers to the relationship that exists between a non-polar particle and a polar particle. As a result of this interaction, the polar particle, which is characterized by a permanent dipole, can induce a temporary dipole in the non-polar particle (Figure 6). Displacement of electrons occurs at a non-polar particle, influenced by an electric field generated by a polar particle [142]. This interaction can be described using the Debye energy Equation (2):

(2) $$E_{D}=\frac{-2{\mu_{ind}}^{2}\gamma }{r^{6}}$$

where $\mu_{ind}$ is the moment of the induced dipole, *r* is the distance between molecules, γ is the polarizability, or a measure of the ability of particles to polarize or deform electron clouds.

**Figure 7:**
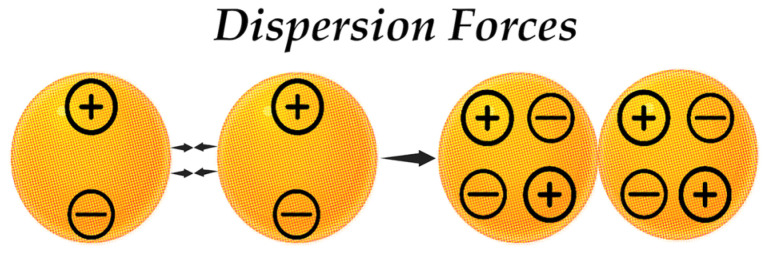
Visualization of the dispersion forces of interaction.

Dispersion forces—also referred to as London forces—act between all particles, irrespective of their polarity, yet they are more characteristic of nonpolar particles (Figure 7). This phenomenon occurs as a result of a temporary fluctuation in the distribution of electrons, leading to the formation of instantaneous dipoles [143]. This interaction can be described using the London energy Equation (3):

(3) $$E_{L}=\frac{-2{\mu_{inst}}^{2}\gamma^{2}}{r^{6}}$$

where $\mu_{inst}$ is the instantaneous dipole moment, *r* is the distance between molecules, γ is the polarizability, or a measure of the ability of particles to polarize or deform electron clouds.

All the above types of intermolecular interactions play a significant role in the formation of such physicochemical properties of colloidal systems as fluidity, viscosity, and stability. In such systems that contain NPs, where both polar and non-polar objects can be found a dependence on all three van der Waals forces is often observed.

#### 4.4.2. Electrostatic Interaction Forces

Electrostatic laws have an important role in the assembly and organization of NPs into various nanostructured materials. Through the regulation of self-assembly processes, these laws enable the formation of 1-D NMs. For example, the spontaneous formation of long chains, similar to pearls, is possible when NPs, in this case CdTe quantum dots, interact with negatively charged thioglycolic acid [144]. In case of non-spherical particles, the electrostatic interactions take on a more complex character. The distribution of the electric field surrounding these particles undergoes alterations, contingent upon their shape. Elongated forms of NPs, such as rods, exhibit more pronounced dipole moments, which can result in side-interactions, including charge-induced dipole interactions [145].

Even though in the case of the nano-world, some physical laws should be revised, in the case of electrostatics, the laws characteristic of macroscopic objects apply here, of course, considering the specifics of the nanoscale.

Coulomb’s law is a foundational principle in electrostatics, elucidating the interaction force between two-point charges on different scales [146]. Coulomb’s law is also applicable for the investigation of the forces of interaction between NPs, even though it was described for macro-objects. The law can be physically described by the well-known Coulomb force Equation (4) [147]:

(4) $$F=k\frac{\left|q_{1}\right|\left|q_{2}\right|}{r^{2}}$$

where *F* is the force of the interaction, N; *k* is the coefficient of proportionality (Coulomb’s constant), equal ~8.99 × 10^9^ N·m^2^/C^2^; *q*_1_ and *q*_2_ are the values of the charges, C; *r* is the distance between charges, in case of NPs measured in nm.

Coulomb’s law quantifies the forces of attraction or repulsion between charged NPs, which is important for understanding the properties of nanostructures in solution and during NMs formation [148]. In the context of colloidal systems, where particles are suspended, the electrostatic interaction forces described by Coulomb’s law assist in preventing their aggregation [149].

### 4.5. Electric Double Layer (EDL)

The formation of EDL is a characteristic feature of NPs in solution. This is a structure that forms near the surface of the particles in solution. The structure itself consists of a layer of counterions—a layer of ions adsorbed on the surface of a charged NPs, neutralizing their charge; and a diffuse layer, which is located behind the layer of counterions, in which the ions are distributed along a concentration gradient, that is, the farther away from the surface of the particle, the fewer the ions [150].

**Figure 8:**
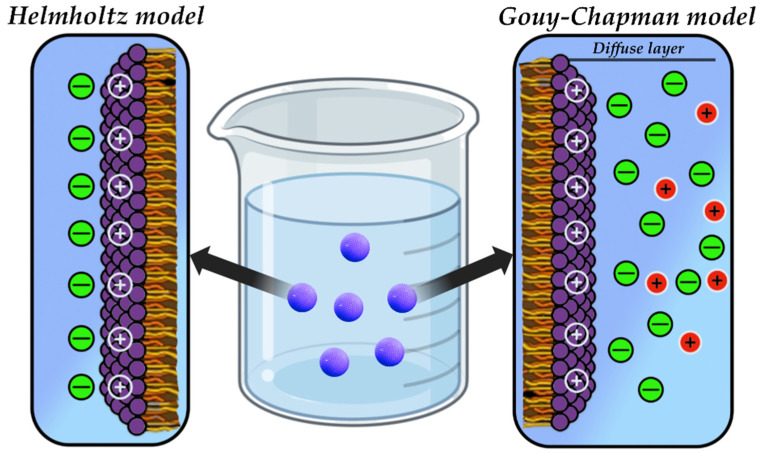
Schematic representation of the Helmholtz and Gouy–Chapman models for describing EDL around NPs surfaces.

There are two main models for the representation of EDL around surfaces, including NPs (Figure 8). The Helmholtz model represents a double layer consisting of a fixed, immobile layer of counterions and a diffusion layer [151]. The model operates under the assumption that the surface charge of the NPs generates an electric field, thereby attracting counterions from the surrounding solution and inducing an electric potential near the surface. However, the Helmholtz model has its limitations, since it does not consider the diffuse nature of ions in solution and rather describes the static state of counterions [152]. This simplification may not be sufficient to describe systems in which the distance between the particles is minimal or the ions have a significant ionic radius. Despite the model has limitations, it has been used as the basis for the more complex Gouy–Chapman model [153,154].

The Gouy–Chapman model represents a more advanced version of the aforementioned model, extending the concept proposed by Helmholtz. It considers the diffuse nature of ions in solution and describes their distribution around the charged surface of the particle (Figure 8) [154]. The Gouy–Chapman EDL structure also contains a fixed layer of counterions and a diffuse layer, but with several differences. Firstly, the diffuse layer is fully incorporated into this model, in contrast to the previous model where it was not considered at all. Secondly, ions in motion are considered [155]. The model describes the electric field that is generated by the charged surface of a particle and will affect on the distribution of ions in the diffuse layer. The Gouy–Chapman model is applied to describe interactions in colloidal systems and systems containing NPs, explaining how electrostatic forces affect the stability of suspensions, particle aggregation and other physicochemical aspects of nanoscale systems [155].

EDL exert influence on both electrostatic interactions and the stability of colloidal systems. In the context of electrostatic interactions between NPs, the relative location to each other is considered. At small distances between particles, EDL can merge, leading to a change in ion distribution and, consequently, affecting the interaction between particles [156]. From the perspective of stability, the predominance of repulsive electrostatic force arising from EDL over the attractive van der Waals forces is pivotal in ensuring stability of the system [157]. Conversely, if the attractive forces are predominant, aggregation of particles and a consequent loss of stability of the system will be observed.

#### 4.5.1. Derjaguin–Landau–Verwey–Overbeek Theory (DLVO)

This cross-theory describes the interactions between colloidal particles, considering both electrostatic and van der Waals forces. From the standpoint of electrostatics, this theory considers the influence of EDL, which was discussed above [157]. In addition to electrostatics, DLVO also takes into account intermolecular van der Waals interactions arising from temporary dipole moments. Combining both aspects of interactions between particles, this theory interprets that colloidal particles of a dispersed system, due to the presence of Brownian motion, can freely approach each other until they come into contact with their diffuse shells or layers, for further convergence, the particles deform their shells for mutual closure [158]. Thus, this theory has become universal in the scientific community, combining aspects of interactions between particles. The formula can describe the force of interaction between NPs using the Derjaguin approximation 5 [157]:

(5) Fd=2πW(d)1R1+1R2

where *R*_1_ and *R*_2_ are radii of two interacting particles, $W_{\left(d\right)}$ is the energy of the interaction between two surfaces.

The DLVO theory makes an indisputable contribution to understanding the interaction of NPs used as targeted drug delivery systems. From a physicochemical perspective, the theory enables precise prediction of particle interactions, primarily with other particles, facilitating assessment of their stability [159]. However, it should be noted that this theory does not consider the behavior of particles in an aggregated state, nor their mechanical properties and is a common cross-theory to understand the main aspects of colloidal stability of particles [160].

#### 4.5.2. Johnson–Kendall–Roberts Theory (JKR)

In contradistinction to DLVO, the JKR theory concerns the mechanical properties of the material and thus describes the interaction between soft and easily deformable objects including adhesion and contact between the NPs [160]. The theory is predicated on short-range adhesive van der Waals forces. JKR theory considers that when particles come into contact, deformation occurs during the interaction of soft objects. For example, the shape and size of the polymeric NPs may change, affecting the adhesive force and the behavior of the colloidal system [161,162]. The calculation of the adhesion force can be presented using Equation (6) [163]:

(6) FA=32πγR

where γ is the separation energy per unit of area, and *R* is the radius of curvature of the object.

JKR theory is a significant instrument for comprehending the interaction between soft particles. Nevertheless, as in the case of DLVO, it is limited by several factors:

- Not applicable for solid objects;
- The particles are assumed to possess a symmetrical shape and uniform force distribution;
- Dynamic factors, such as the rate of convergence of particles and the influence of external forces, are not taken into consideration.

#### 4.5.3. Derjaguin–Muller–Toporov Theory (DMT)

In comparison with JKR, DMT theory describes the interaction between solid particles based on weak, long-range forces, making it suited for describing NPs contact with minimal, insignificant deformation [164]. The basic equation of DMT theory for the adhesion force (*F_A_*) can be presented as follows [164]:

(7) $$F_{A}=2\pi R\varphi$$

where *R* is the radius of the spherical particles, φ is the energy of the interaction per unit of area.

The adhesion force depends on the radius of the particles and their surface energy, as well as the distance between them. As the distance between objects increases, the adhesion force will decrease [164]. As with other theories, DMT is limited and describes processes that are characteristic of certain physical objects [160].

However, all three of the theories are fundamental for understanding the physical processes of the interaction of NPs with each other and complement each other [165,166]. The DLVO, JKR and DMT theories are of particular significance in the development of universal targeted agents for the delivery of pharmaceuticals and NMs for therapies of various genesis.

### 4.6. Surface Plasmon Resonance

A unique electron-optical phenomenon—localized surface plasmon resonance (LSPR)—is observed on the surface of inorganic NPs, namely metal particles [167]. This physical phenomenon causes a number of unique optical properties of NPs, including photoluminescence [168]. The red and blue shift effect, i.e., the dependence of absorption in the UV-visible range on size, is a consequence of plasmon resonance in metallic nanostructures [169]. The dependence of the LSPR distribution and intensity on the shape of the particles is also known [170].

**Figure 9:**
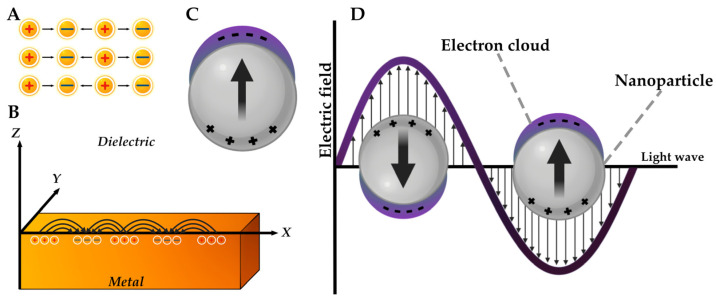
Graphical visualization of plasmon types, where (**A**) is a bulk plasmon, (**B**) is a surface–propagating plasmon, (**C**) is a plasmon localized on the surface, and (**D**) is a visualization of the phenomenon of LSPR in NPs (adapted from [171]).

This phenomenon occurs when the frequency of the incident photon is kept constant during the collective excitation of the conducting electrons, which are known as plasmons [168]. From a physical perspective, light (i.e., a form of optical oscillation) is constituted by photons. In turn, a plasma oscillation is comprised of quanta, i.e., plasmons. The following types of plasmons are distinguished: bulk, surface–propagating and surface-localized (Figure 9) [167]. The longitudinal nature of bulk plasmons results in their insensitivity to visible light excitation. The second type of surface plasmon is characterized by its propagation over the surface of a metallic materials and possesses a wave-like nature [167]. Previously, LSPR was described only for metallic NPs; however, one of the articles describes that this phenomenon is also characteristic of particles of a different nature, describing modern nanostructured objects in pharmacy as nanoantennas [126].

In the field of medicine, LSPR of metal particles has been applied as an optical sensor for the detection of chemical compounds and biomolecules. The application of AgNPs as a tool for the quantitative determination of drugs using oxymetazoline and trenbolone acetate as examples was demonstrated [172,173]. This phenomenon was also applied as a biosensor for the diagnosis of neurocysticercosis, where *Taenia solium* antigens were used as a model [174]. In the work of Mahmudin L. et al., the use of the AgNPs LSPR phenomenon as a biosensor for *Escherichia coli* is described [175]. Plasmon resonance has also been demonstrated to be a useful tool for the detection of a range of biomolecules. By analyzing the optical properties of AuNPs and AgNPs, scientists have concluded that the LSPR phenomenon is a potential biosensor for both nucleic acids and other biological structures [176].

### 4.7. The Phenomenon of Intrinsic Radiothermal Emission

**Figure 10:**
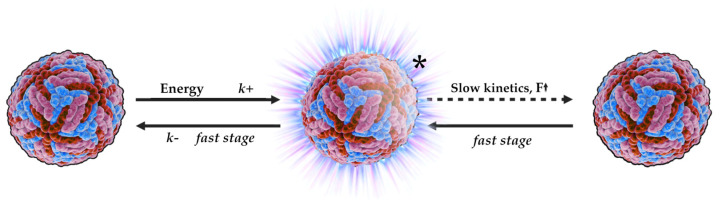
Visualization of the kinetic scheme of the formation of quasi-plasma regions on the surface of NPs, where F is the flux density of intrinsic radiothermal emission, and * is an activated form of NP (adapted from [126]).

Recently, the physicochemical property of NPs based on their increased electric field strength has been described [126,177]. This property has been demonstrated to contribute to the formation of quasi-plasma regions on the surface of the particles (Figure 10). The formation of these regions is described by the following kinetic scheme:

Following the absorption of energy by the NP, a metastable activated NP* state is generated. This metastable state can rapidly revert to its original inactive state or gradually transition to the NP state. The latter process is accompanied by the stabilization of the dipole and the formation of a quasi-plasma region.

The present scheme indicates that slow supramolecular NP transitions are a source of radiothermal emission.

To comprehend the essence of this phenomenon, it is worth to initiate the discussion with the forces of interaction of NPs with each other. In the aqueous solutions or in the forms with a high-water content, the particles enter van der Waals interactions according to the “surface to surface” principle [178,179]. As illustrated in Figure 10, the transition from an excited state to an inert state of NPs is characterized by slow relaxation kinetics, attributable to the conformational mobility of particles [180]. In the case of irregularly shaped particles interacting with each other, they are polarized, which in turn leads to an increased electric field strength, which can form certain plasma-like areas or electron clouds on the surface of the NPs [126]. These plasma-like areas will be the source of the radiothermal emission.

This physical phenomenon has found a response in the quality control of drugs containing biologically active NPs. The quality control of immunobiological pharmaceuticals such as interferons and VLP vaccines were described [177]. The article demonstrates that the detection of radiothermal emission can be used to determine the qualitative characteristics of the pharmaceuticals. The article further describes an experimental method of immunization control based on a laboratory animal model. Through a comparative analysis of the emission results and immunological analysis, it was demonstrated that during the process of immune response formation to the administered vaccine, specifically cell-mediated immunity, the emission from immunized experimental subjects surpasses that of the control group [177].

### 4.8. Magnetic Properties of NPs

All the above-mentioned physical properties of NPs are of importance to comprehend their application in the fields of pharmacy, medicine, and biology. However, ultra-small particles possess additional physical properties.

The majority of inorganic NPs, specifically metal particles, are characterized by their magnetic and thermal properties as previously outlined, the transition to the nanoscale range gives rise to the manifestation of quantum effects, namely quantum limitation [181]. It is important to note that not all metal NPs possess magnetic properties. It is logical to hypothesize that particles containing magnetic elements will exhibit these properties. Diamagnets, which include Au and Ag, do not exhibit magnetic properties. Quantum limitation, in turn, affects these properties, causing an effect such as superparamagnetism [182], i.e., the occurrence of magnetic phenomena only in the presence of an external magnetic field [183]. Superparamagnetism is a size-dependent property. As the particle size decreases, the energy of magnetic anisotropy per particle will decrease [184]. In the field of medicine and pharmacy, this property finds application in the magnetic delivery of drugs [185,186].

### 4.9. Thermal Properties of NPs

The effect of the NPs ultra-small size on their thermal conductivity is significant. As the size of the particles decreases, the ratio of their surface area to their volume increases exponentially [19,187]. It is evident that an increased surface-to-volume ratio will provide a greater number of electrons for heat transfer. Furthermore, micro convection, which occurs as a result of Brownian motion when solid particles enter a liquid medium, will also contribute to thermal conductivity in the NPs [188]. Quantum effects also affect the thermal properties of particles. The Gibbs–Thomson effect demonstrates that as particle size changes and moves into the nano range, the melting point also changes, which is related to the number of available energy levels [189]. The application of metal NPs in photothermotherapy is well known. The photothermotherapy process involves the accumulation of AuNPs in the tumor area, followed by laser irradiation, which results in the destruction of cancer cells [190,191].

## 5. Methods of NPs Synthesis

There is a multitude of contemporary approaches to the production of NPs that are contingent upon their nature of origin. These methods can encompass conventional chemical synthesis via sequential reactions or more intricate procedures involving recombinant systems. This section will explore the most frequently employed techniques to produce NPs, which were mentioned in this review.

### 5.1. Synthesis of LNPs

**Figure 11:**
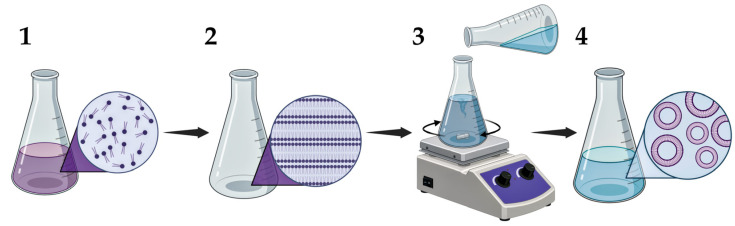
Schematic visualization of LNPs synthesis by the thin-film method, where 1—mixture of lipids dissolved in organic solvent; 2—formation of thin lipid films on the walls of the flask after the organic solvent evaporation stage; 3—addition of aqueous medium with stirring to the flask with formed thin lipid films; 4—formation of LNPs of different sizes after the hydration stage.

There are several methods to produce liposomes. The first known method for the synthesis of LNPs was discovered by Alec Bangham [48]. The thin-film method involves dissolving lipids, as described for phospholipids, in an organic solvent, forming a thin film after the solvent evaporates (Figure 11) [192]. When a buffer solution is added, hydration of this film occurs, resulting in the formation of liposomes. However, despite the simplicity of the above method, there are several difficulties in its application, one of which is the heterogeneity of the liposomes obtained [192].

**Figure 12:**
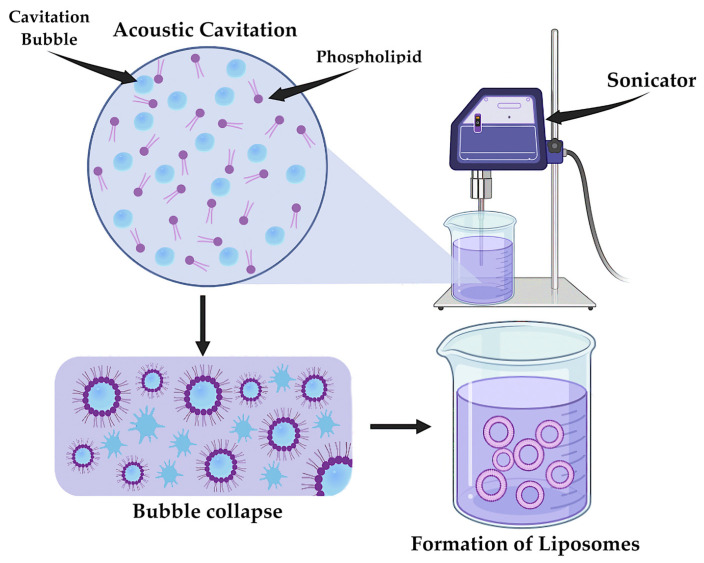
Schematic visualization of LNPs synthesis by the sonication method.

An alternative method for the synthesis of LNPs is sonication (Figure 12) [193]. Ultrasonic waves create cavitation in the lipid-containing solution, resulting in the formation of microbubbles [194]. The process of synthesizing LNPs includes the rapid collapse of these bubbles, causing an increase in temperature and pressure. The result is the destruction of lipid films and their mixing with the aqueous phase, leading to the formation of LNPs. Compared to the previous method of liposome synthesis, this method allows control of the size characteristics of the particles obtained, which may have a positive effect on the pharmacokinetic variability of the final drug [195].

The most widespread and modern method of LNPs synthesis is the microfluidic extrusion method. The method is based on infusing a lipid solution through a microfluidic device, where the lipid films are disrupted by passing through narrow channels due to high pressure and flow velocity, with subsequent formation of liposomes [196]. This method allows LNPs with specific size and morphological characteristics to be obtained at the outcome [197].

### 5.2. Biosynthesis of VLP

**Figure 13:**
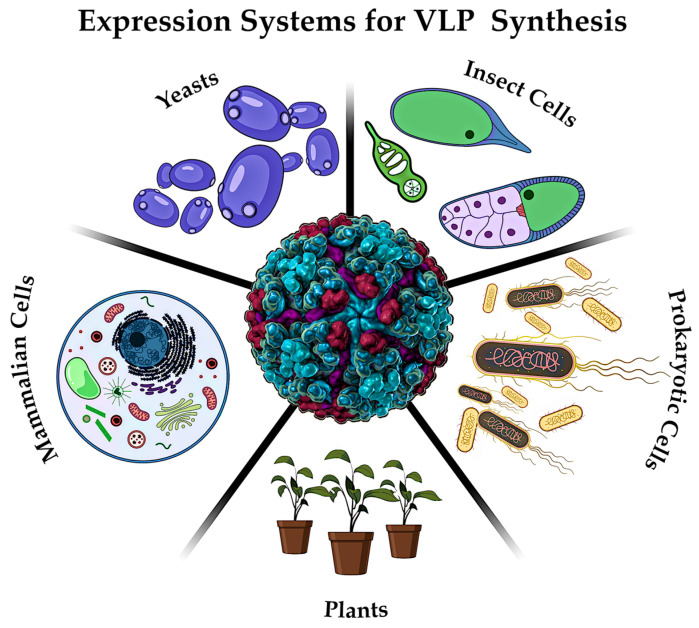
Types of expression systems applied to produce recombinant viral proteins for VLP synthesis.

The synthesis of VLP particles is frequently accomplished through the application of diverse expression systems (Figure 13), which are imperative to produce recombinant viral proteins [198]. These systems can encompass yeast [199], insect cells [30], prokaryotic cells [200], plants [198] and mammalian cell lines [201]. The predominant distinction among these systems pertains to the capacity for post-translational modifications, which are deemed essential for the optimal functionality of finalized VLP vaccines.

For example, bacterial systems based on *Escherichia coli* do not permit subsequent modification of the obtained protein, while in the case of the insect system it is possible to carry out rather complex posttranslational modifications [202]. Despite the differences, all the above systems are relevant and are used for the synthesis of viral proteins. The subsequent steps of VLP synthesis include protein folding, isolation and stabilization [30].

### 5.3. Synthesis of Polymeric NPs

**Figure 14:**
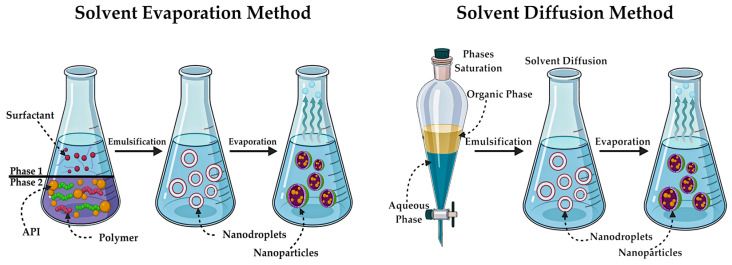
Schematic visualization of the synthesis of polymeric NPs by solvent evaporation and solvent diffusion methods, where Phase 1 is an aqueous phase containing surfactants and Phase 2 is an organic phase containing polymers (red and green structures) and APIs.

The synthesis of these particles is usually associated with the production of oil-in-water emulsion. There are various methods for the preparation of polymeric nanospheres and nanocapsules. The most common is the solvent evaporation method, where emulsion preparation is also employed (Figure 14). The polymer and API are integrated into the organic phase, followed by emulsification with the aqueous phase, which contains the surfactant in addition to water. This process eventually forms nanodroplets [203]. Subsequent to obtaining the finished emulsion, the solvent is evaporated, resulting in polymer precipitation and the formation of NPs [77].

The solvent diffusion method is analogous in principle to the previous one. The diffusion of solvent from the dispersed droplets, formed during emulsion formation, into the aqueous phase (Figure 14), leads to the precipitation of the polymer [204]. This method allows for the precise control of particle size [205].

**Figure 15:**
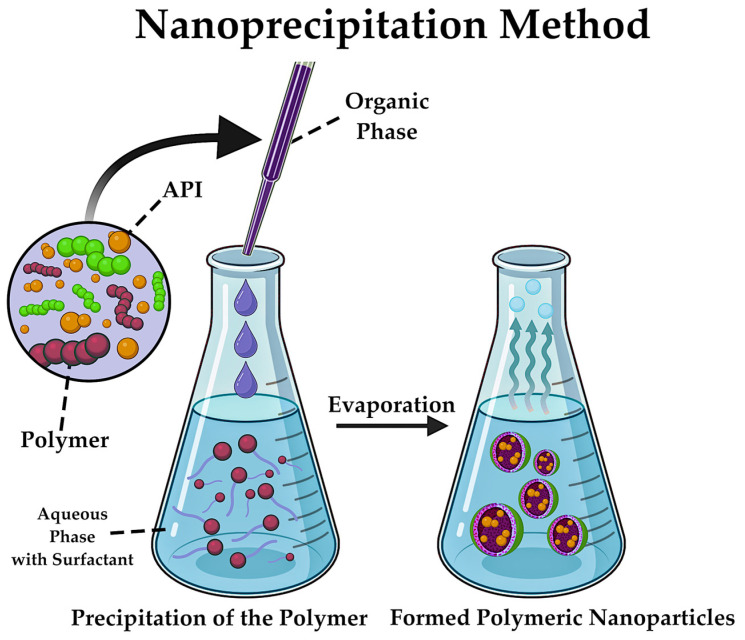
Schematic visualization of the synthesis of polymeric NPs by nanoprecipitation, where the organic phase contains polymers (red and green structures) and APIs and the aqueous phase contains surfactants.

Nanoprecipitation is regarded as one of the most accessible methods in terms of equipment. This method is based on the precipitation of polymer from solution after displacement of organic solvent from the lipophilic solution into the aqueous phase [77]. The nanoprecipitation method involves the controlled integration of the organic phase containing the polymer and active substance into the aqueous phase, leading to a decrease in polymer solubility and the rapid diffusion of the polymer solution into the aqueous phase, resulting in the formation of NPs (Figure 15) [206]. The morphological characteristics of the final product are contingent on several factors, including the rate of integration of the organic phase, the concentration of polymer and/or API [207]. Due to the flexibility of the process, it is possible to achieve the necessary dimensional characteristics for NPs by varying the synthesis conditions.

In addition to the aforementioned methods, scientists are actively employing alternative techniques for synthesizing polymer nanostructures that do not necessitate an emulsification process. The study of the catalytic abilities of intracellular enzymes has been a source of inspiration for many researchers, who have created nanostructures that operate on a similar principle [208]. Nonetheless, the process of synthesizing these enzyme complexes is intricate, primarily due to the necessity of identifying the most appropriate method for their synthesis. It is evident that the complexity inherent in the control of the spatial distribution and number of active enzymes is a major challenge [209,210].

The authors of one article proposed a methodology for producing enzyme complexes based on polymer nanocapsules [210]. This delivery system has been demonstrated to be both more efficient and stable. The synthesis of these polymer nanocapsules is based on the conjugation of enzymes with single-stranded DNA and subsequent encapsulation in polymer capsules. The results of the study demonstrated increased catalytic efficiency and improved stability of the obtained structures, as well as the possibility of leveling toxic intermediates formed during enzymatic reactions [210]. These results are indicative of positive dynamics for the use of these particles for medical and pharmaceutical purposes.

The present study proposes a contemporary approach to the synthesis of enzyme nanocomplexes based on polymer NPs [78]. The approach to the synthesis of polymer nanocapsules, as demonstrated by the authors, is based on the introduction of polymerizing acrylic groups and radical polymerization. Polymerization, in addition to creating a polymer shell, plays a role in stabilizing protein structures. The use of three different monomers has enhanced the physicochemical properties of the obtained objects, leading to improved control of the interaction of capsules with other molecules and cells in vivo [78].

### 5.4. Synthesis of Dendrimer NPs

Even though dendrimers are polymer-like structures, it is worth considering the methods of their synthesis separately. The synthesis of dendrimer structures involves chemical reactions, and the methods of their preparation are divided into two categories: divergent and convergent. Divergent synthesis is a process in which layers of a dendrimer structure are built up around a central link by multiple repetition of a sequence of chemical reactions [211]. The convergent method bears resemblance to the previously outlined approach, with the distinction lying in the initial stage of convergent synthesis, where dendrons, serving as “building blocks” are obtained and subsequently attached to the functional core [212].

In the contemporary scientific community, both synthesis methods are used with various modifications. The authors obtained dendrimers of the 3rd generation, that is medium-size structures with an internal space, by repeating the chemical reactions of Passerini and by cross-metathesis of olefins followed by hydrogenation and hydrolysis [213].

Applying the aforementioned Passerini reaction, the team of scientists describes in detail a convergent method for the synthesis of surface-triblock dendrimers [214]. However, despite the prevalence of these methods, some researchers have noted several disadvantages and limitations in using divergent and convergent approaches [215,216]. In the aforementioned works, the authors observe the employment of a novel approach for the acquisition of dendritic structures, namely click chemistry, and provide an evaluation of its efficacy.

### 5.5. Preparation of Inorganic NPs

Metal NPs can be synthesized through a variety of methods, including chemical, physical, and biological approaches. The most prevalent chemical method for producing metal NPs is the reduction reaction. As outlined in the research [217], this approach involves the reduction of Au^3+^ to Au^0^, resulting in the formation of AuNPs with a size range of up to 20 nm [217]. The researchers’ chosen approach is one of the modifications of the Brust-Schiffrin method [218] for the synthesis of metal NPs, which allows to obtain particles of a strictly defined size [219].

Physical methods encompass mechanical grinding, i.e., the fragmentation of large materials to nm size [220]; evaporation and condensation, where laser ablation is employed to vaporize the material [221] and plasma is applied to condense NPs [222]; and electro-explosion, which involves the final condensation of atoms into particles [223].

Whilst all the aforementioned methods allow to produce homogeneous NPs of a defined size, concerns regarding the environmental impact of these processes frequently feature in scientific literature. In response to these concerns, a significant number of studies have been conducted with the aim of developing a modern, environmentally friendly approach to particle production, in line with the popular trend of green chemistry. Several review articles demonstrate a range of modern techniques for synthesizing AuNPs and AgNPs based on bacteria, plants, and fungi [224,225]. The authors also touch upon the ways of application of the obtained biosynthetic particles, noting the positive dynamics in drug delivery, their reduced toxicity, as well as the flexibility of their design [224].

The synthesis of silicon nanostructures is characterized by a range of physicochemical methods, the selection of which is contingent on the desired type of silicon NPs. The focus of scientific papers is twofold in non-porous and mesoporous silicon NPs. The following physicochemical synthesis methods are characteristic of non-porous particles: sol-gel method [226], microemulsion synthesis [227], Stöber synthesis method [228], precipitation reactions [229], etc. In the context of mesoporous particles, the enhanced Stöber method [230], self-assembly induced by evaporation [231], and the co-condensation method [78] and a number of other methods are employed.

### 5.6. Synthesis of Carbon NPs and Quantum Dots

The last NPs synthesis methods to be examined in this review are for carbon particles and quantum dots. As outlined above, the most notable examples of carbon nanostructures are fullerenes, which vary in the number of carbon atoms they contain. The most canonical fullerene is believed to be fullerene C_60_, which was discovered in 1985 by a group of scientists using mass spectrometry to study graphite vapor [232]. Other carbon particle structures are currently known, such as C_70_, C_84_, C_120_, etc. [233]. For their synthesis, the physical methods that were mentioned in the previous paragraph are more commonly used, with the most common method to produce fullerenes being laser ablation [234]. A number of chemical routes for their production are also known. The authors of the article described a method for obtaining various monoadductive variations of fullerene C_60_ with antiproliferative activity [235]. This method involves the cycloaddition reaction of precursors of fullerene derivatives.

In addition to fullerenes, which are the most well-known carbon-based NPs, there are other carbon NMs that are being applied in pharmacy. Carbon nanotubes, which are 1-D NMs, at this stage of their development are a promising platform for theranostics, pharmaceutical quality control and targeted drug delivery [236,237]. The fabrication of these nanostructures is typically achieved through a range of techniques, including arc discharge, laser ablation, or chemical vapor deposition [238,239].

In recent years, the application of quantum dots in pharmacy and medicine has been actively developed. Continuing the theme of carbon-based NMs, it is worth noting that there are both carbon-based quantum dots [240] and, more familiar to the scientific world, semiconductor-based quantum dots [241,242,243]. The synthesis of carbon quantum dots is achieved through the application of various physicochemical methods. The most common methods are laser ablation [244], electrochemical oxidation [245] and sol-gel [246], synthesis in colloidal solutions (aqueous and non-aqueous) [246,247], etc. The process of chemical synthesis necessitates a series of reactions, including carbonation, pyrolysis, and oxidation [248]. In addition to the inherent risks associated with the utilization of hazardous chemicals in the production of quantum dots, scientists are turning to green synthesis [249].

## 6. Contemporary Approaches to NPs Modification

The physicochemical properties of NPs, including surface composition, superficial charge, size, and shape, are considered to be the key factors that affect the biocompatibility and uptake efficiency of nanoplatforms.

**Figure 16:**
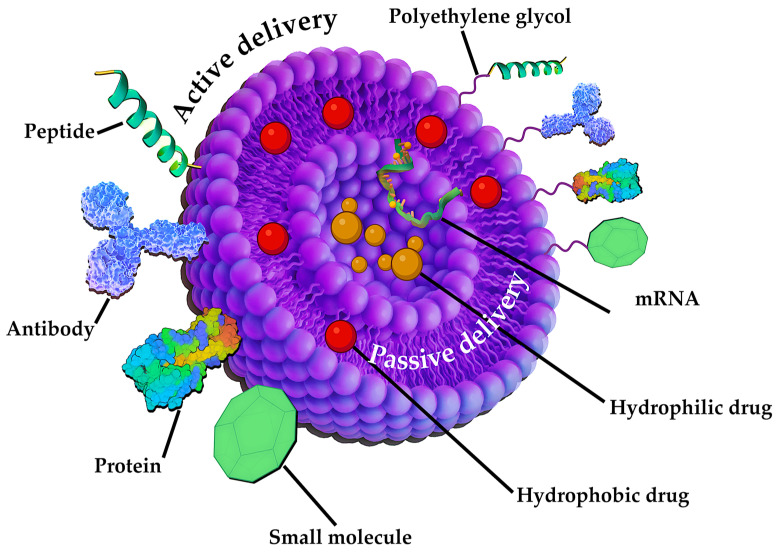
Active and passive drug delivery methods by LNPs as a representative example. Visualization of active (integration of specific ligands on the surface of liposomes) and passive (encapsulation of drug substance inside LNPs) delivery of API.

For example, the following articles describe various pharmaceutical integration methods using liposomes as a case in point [250,251]. The authors distinguish two methods—active and passive. The first is bringing the liposomes to the “magic bullet” concept presented by Paul Ehrlich in 1906 [252]. This principle of drug delivery involves the use of various mechanisms such as the integration of specific ligands (antibodies, protein structures, peptides, etc.) [253], which leads to a more efficient accumulation in cancer cells (Figure 16). On the other hand, the passive method of drug transport excludes the modification of LNPs and is carried out by simply encapsulating different pharmaceuticals into the liposomal core and delivering them through increased permeability, allowing them to accumulate in tumor tissues [254].

The aforementioned approaches to drug delivery are applicable not only to liposomes, but also to a broad spectrum of other NPs. The mechanism of active targeting is based on highly specific and efficient delivery of nanostructures to specific cells, avoiding non-specific binding and accumulation of particles in normal tissues [255]. Conversely, passive targeting is based on the effect of enhanced permeability, allowing nanocarriers to penetrate various organism barriers and accumulate at the site of action [256].

Active targeting is of the most significant scientific interest, as indicated by data obtained from PubMed. Therefore, it is worth considering in more detail the various approaches to modifying NPs surfaces. One of the first phase of surface modification is based on the use of homo- or heterobifunctional crosslinking agents in order to add an organic functional group (R-NH_2_, R-COOH, etc.) [257]. This methodology facilitates the effective binding of biological molecules to NPs. Polyethylene glycol, in the synthesis of which specific end groups can be selected, has been demonstrated to function as homobifunctional and heterobifunctional linkers for the modification of various nanostructures [258]. In the context of silicon dioxide NPs, aminosilanes are the most widely employed linkers. These linkers introduce an amino group onto the surface of the NPs, thereby facilitating subsequent bioconjugation [259,260]. Mesoporous silicon particles are characterized by functionalization with carboxylic, thiol, and amino groups, which in turn improves the drug-loading capacity [261]. The functionalization of noble metals, such as Au, can be achieved through the application of crosslinking agents that possess -SH or -NH_2_ groups. These groups possess the capacity to react with the metal, thereby forming a covalent bond [262,263]. The modification of metal oxides can be facilitated by the incorporation of functional groups, including amine, carboxylic acid, and analogous moieties [264,265]. The oxidation process enables the generation of -COOH, -OH, and -C = O groups on the surface of carbon-based NPs [264,266].

Also, modification of the NPs surface can be achieved using two different approaches: non-covalent and covalent conjugation. Typically, non-covalent interactions are used to load nanostructures with molecules to be released in target cells such as drugs, while covalent bonds are used to bind ligands useful for targeting and/or reducing NPs toxicity.

Antibodies are often used to modify the surface [267,268,269] (Figure 16). The aforementioned covalent and non-covalent approaches are frequently employed for the conjugation of antibodies to the surface of NPs. The experimental work describes the production of NPs modified with antibodies specific to the human epidermal growth factor 2 receptor by non-covalent binding [267]. In a review paper devoted to the search to identify an effective non-covalent binding of antibodies to NPs, the strategy of interaction of antibodies with pegylated particles is touched upon [270]. The authors observe that this approach results in a more stable specific binding of antibodies, which can have a positive effect on targeting tumor tissues and their internalization. Employing the principle of nucleophilic aromatic substitution, the researchers proposed an approach for the covalent coupling of antibodies with iron oxide magnetic particles [265].

In addition to antibodies, a contemporary approach to the modification of NPs that merits attention involves the utilization of diverse peptide variations (Figure 16). Numerous NPs modifications incorporating peptides have been documented in the scientific literature. For example, to enhance the bioavailability and targeted delivery of drugs for chronic kidney disease, the authors have developed small organic NPs—peptide amphiphilic micelles [271]. The review paper examines the most effective approaches to modifying the surface of various particles with peptides to enhance their permeation through the blood–brain barrier [272].

The modification of nanostructures by peptides, as well as by other biologically active molecules, occurs through conjugation with the surface of particles [273]. A variety of “grafting” approaches are utilized in the preparation of peptides intended for conjugation to the surface of particles. These approaches encompass “grafting to” and “grafting from” techniques, which are employed in the synthesis process [273]. In the case of “grafting to”, a number of specific reactions with terminal or side groups are carried out, namely the nucleophilic attack of amino groups [274], “click” reaction of azide with alkyne [275] or the thiol-ene binding of cysteine residues to alkene-containing groups [276]. In contrast to the previously described approach, the “grafting from” method involves the introduction of a ligand at one of the initial stages of the synthesis of the peptide structure [272].

In addition, it is important to acknowledge the extant methodologies employed in the synthesis of peptide structures. The most common method of synthesis is known as solid-phase peptide synthesis. It was first described in 1963 by R.B. Merrifield [277]. Merrifield was awarded the Nobel Prize in 1984 in recognition of this achievement. This method is based on the presence of a solid matrix in the form of a polymer, which serves as a kind of support for the construction of the peptide chain [278]. This method finds application in the pharmaceuticals area for the automated synthesis of peptide chains with specific properties. In addition to the fundamental solid-phase synthesis, there are others such as liquid-phase [279], chemical [280] and recombinant synthesis [281]. Each of these methods offer advanced possibilities for peptide synthesis, including the production of polypeptide chains, the integration of additional protecting groups, and green synthesis based on bacteria and yeast, which significantly enhances the environmental friendliness of peptide production.

## 7. Contemporary Application of NPs

Following a comprehensive examination of contemporary methodologies for synthesizing and modifying NPs of various natures, it is important to direct attention to a significant aspect of this review, namely their application in pharmacy and medicine.

### 7.1. Application of LNPs in Pharmacy

The first mention of compounds encapsulated in liposomes dates to 1974 [282,283]. The study of the possibility of encapsulating various APIs continues to this day, and the variety of pharmacological groups proposed for targeted delivery never ceases to amaze.

The most frequently active substance encapsulated in liposomes and applicated in anticancer therapy is doxorubicin, as evidenced by the extant literature. Paolino et al. [284] described the possibility of modifying LNPs containing doxorubicin, thereby increasing its pharmacokinetic parameters and reducing its side effects. The employment of “super-stealth-liposomes”, that incorporates polyethylene glycol onto the surface of LNPs, has been demonstrated to enhance the preclinical efficacy of this NPs in the context of breast cancer metastasis [284].

Despite the significant number of publications concerning LNPs in the field of oncotherapy, these particles also find application in immunology. The global pandemic of SARS-CoV-2 compelled prominent pharmaceutical companies to rapidly synthesize vaccines, a process in which LNPs played a major role. It is noteworthy to mention the vaccines that were introduced to the market in those years by Pfizer and Moderna, both of which applied the LNPs model as the mRNA delivery system [285]. In addition to this, a plethora of other immunobiological pharmaceuticals have been developed, applicating liposomal particles for the prevention and treatment of various infectious diseases, including the Zika virus [286], tuberculosis [287], and influenza virus [288].

### 7.2. Application of Modern Nanovaccines—VLPs

**Figure 17:**
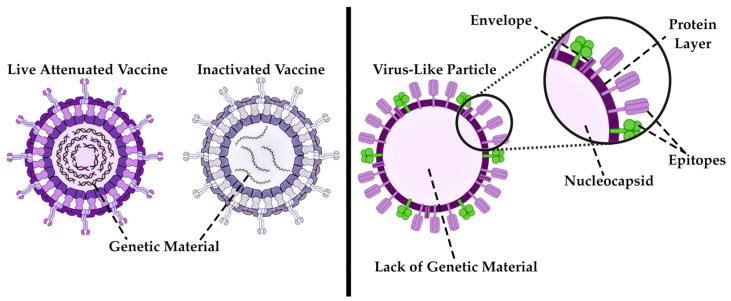
Comparisons of live attenuated and inactivated vaccines with VLP.

Modern immunobiological pharmaceuticals—vaccines, which are designed to provoke a preventative immune response in the organism to viral infections, have undergone a multitude of modifications over the past decades [289]. The development of diverse vaccine synthesis approaches has enabled scientists to achieve a remarkable success by excluding viral genetic materials from immunological pharmaceuticals (Figure 17), thereby minimizing the risk of side effects [202]. The most significant and progressive developments in immunology and virology have been the production of VLP-based vaccines.

These distinctive immunogenic nanostructures comprise assembled viral proteins or epitopes that play a pivotal role in the antigen-antibody response. Notably, these particles are devoid of viral genetic material, thus rendering them a safer alternative to live attenuated or inactivated vaccines (Figure 17).

As previously outlined, the origins of the identification of the first VLP vaccines can be traced to the early 1970s [60]. However, at that time, their biological nature remained uncharted territory for the scientific community. Through subsequent research endeavors, scientists have demonstrated that the viral capsid possesses the capacity to function as a material for the formation of viral particles [201].

VLPs themselves are distinguished from one another by virtue of their structural characteristics, which manifest as either an icosahedral or rod-like shape [290]. The size spectrum of these particles extends to the nanoscale range, with a measurement interval ranging from 20 to 200 nm [126,291]. This enables the potential for the application of these NPs as an effective delivery system to lymphatic organs, thereby facilitating successful activation of the immune response [292].

The primary application for VLPs is, naturally, their use as a prophylactic measure against viral diseases. Currently, VLP vaccines have been developed against a number of pathogens, including the Hepatitis B virus, *Plasmodium falciparum*, Ebola, Rotavirus, SARS-CoV-2, and the HIV, amongst others [293]. In addition to this, there are other notable applications of VLP in pharmacy. A review article discusses the possibility of using viral particles as an effective system for the delivery of APIs, a topic which is of interest to scientists due to the size, encapsulation capability and enhanced biocompatibility of VLPs, meaning they can be used in the most complex pharmaceutical applications, including therapeutic vaccines aimed at aiding cancer therapy [294]. Thus, researchers describe their way to production of a personalized cancer vaccine based on VLPs [295]. The scientists have proposed a method of creating a cancer vaccine based on a complex of neoantigenic peptides, i.e., peptides formed during non-synonymous mutations in the DNA of tumor cells, and VLP. In fact, viral particles bound covalently to neoantigens promote their more efficient presentation to T-cells, which enhances the anti-tumor immune response [296]. This approach underscores the potential of personalized medicine as a novel paradigm in the fields of pharmacy and medicine. In the near future, as the researchers write, it will make it possible to produce the necessary drug right at the patient’s bedside [296].

### 7.3. Applications of Polymeric NPs and Dendrimers

The final representatives of the organic NPs to be discussed in this review are polymeric particles. In addition to the previously discussed objects, they represent a range of modern and unique nano-tools for applied pharmaceutics. The ability of polymeric NPs to improve bioavailability and pharmacokinetic parameters of drugs, as well as to modify physicochemical properties of APIs, allows them to be used to solve complex pharmaceutical problems [77].

As previously referenced, these particles serve as effective drug delivery systems, with numerous studies focusing on the targeted delivery of drugs from diverse pharmacological categories by encapsulating them within polymeric NPs [297,298,299]. However, the most intriguing research endeavors pertain to efforts to modify the conventional drug administration process into the patient’s organism, with the objective of enhancing the quality of life. Researchers developed an oral IgG delivery system based on polymeric NPs, with the results being a careful selection of components for the synthesis of polymeric particles suitable for the preservation of antibodies during oral administration, as well as for their release [206]. A similar idea was shared by scientists, who proposed an alternative delivery of a drug with low bioavailability—insulin [300,301]. It is a well-known fact that, in the modern pharmaceutical market, pancreatic hormone pharmaceuticals are presented in the form of subcutaneous dosage forms, which is not very convenient for patients in terms of daily use. The best alternative would be oral administration, but at the moment this is not possible due to the low stability of insulin in the gastrointestinal tract and low intestinal permeability. However, recent scientific research has demonstrated the potential of polymeric NPs as a delivery system for insulin. These particles have been shown to protect the insulin from enzymatic degradation in the stomach, thereby enhancing its absorption in the intestine [300,301]. Indeed, in such applications, the amount of insulin required is significantly higher than that required for parenteral administration [301]. This field of research is ongoing, scientific groups proposing various methods for synthesizing transport-NPs for insulin pharmaceuticals on different polymers [302]. The results of preclinical animal trials have also demonstrated positive outcomes [303].

Dendrimers, which are polymer-like nanostructures, find their application in pharmacy as a target delivery of drugs of various pharmacological groups. The theranostic approach of using these materials in oncotherapy is described in work [304]. A review paper describes a promising direction of applying dendrimeric prodrugs as delivery systems for APIs, indicating their ability to modify pharmacokinetic and pharmacodynamic properties, as well as to reduce the toxicity of the final pharmaceutical [305]. Zhao et al. investigated the potential use of isotretinoin, a drug for the treatment of skin diseases, conjugated to dendrimers [306]. The authors marked controlled release of the drug as well as improved transdermal penetration of the pharmaceutical through the skin, indicating the effectiveness of applying this combination of the drug with nanostructured dendrimer materials.

### 7.4. Application of Inorganic NPs

Inorganic NPs, namely metal and silica particles, have found success in pharmacy and medicine due to their unique physical properties and simpler approach to their synthesis. AuNPs, AgNPs and mesoporous silica particles are most often used [307,308,309].

The main purpose of metallic NPs in pharmacy and medicine is to efficiently deliver active ingredients in the organism. In this area, particular interest is represented by various developments aimed at improving the lives of patients undergoing oncological therapy. AuNPs are used as nano-instruments for selective targeted delivery of chemotherapeutic drugs [310]. In the presented work, the team of authors describes the use of AuNPs functionalized with monoclonal antibodies in the treatment of prostate cancer, noting the prospects of their use [311]. A highly efficient strategy for targeted delivery of anti-cancer drugs is magnetic targeted delivery. The objective of the subsequent scientific article was to develop biocompatible magnetic NPs based on Au that had been functionalized with polyethylene glycol for the effective loading of doxorubicin [312]. In vivo studies have demonstrated the increased pharmacological efficacy of the magnetic delivery method, thereby minimizing both the random distribution of the antitumour drug and the risk of side effects to healthy tissues. The subsequent study investigates the potential of AuNPs as a means of delivering therapeutic agents for the treatment of pulmonary diseases [313]. The authors of the article observe the disadvantages of NP spraying and present a more effective method of directional particle spraying after endotracheal implantation, noting the dose-dependent two-way distribution of AuNPs without side effects.

APIs transport is not the only application of AuNPs. Their use as imaging agents has been repeatedly mentioned in several articles [314,315]. The modification of metallic NPs to serve as biosensors facilitates the early diagnosis of various diseases [316]. The unique optical properties of AuNPs and AgNPs, such as LSPR, combined with photosensitizing agents, exhibit a synergistic effect, enabling effective phototherapy [317]. These diverse applications of metallic particles position them as a novel class of theranostic drugs [314].

The most canonical methods of application have been considered. Now let’s direct our attention to equally promising variations of metal NPs application in pharmacy. In this review, the enhanced antibacterial activity of inorganic particles was previously discussed [90]. Their synergistic effect with various antibiotic agents allows effective action against both Gram-positive and Gram-negative bacteria [318], as well as reduction of the prescribed dose of antibiotics, thereby counteracting against rapidly developing resistance to pharmaceuticals [94].

Mesoporous silica particles are considered to be a representative of the class of inorganic NPs. Due to their structural characteristics and unique physicochemical properties, including the capacity for enhanced cargo transportation, high biocompatibility, and stability, they are considered as an effective candidates for targeted drug delivery [319]. A number of review papers confirm their effectiveness as targeted agents in the treatment of various diseases [319,320,321].

The administration of certain active agents systemically is rendered impractical by their short half-life and acute toxicity. An example of one of these agents is described in the following article [322]. Tumor necrosis factor α is a cytokine that functions as an immunostimulating agent, inducing tumor necrosis and regression. As the authors themselves assert, the introduction of the substance into the patient’s body is both ineffective and dangerous. However, the employment of dendritic mesoporous silicon NPs enabled the authors to achieve enhanced delivery of the antitumor agent, accompanied by a two-fold reduction in the semi-maximal effective concentration. This, in turn, led to a significant reduction in side effects [322].

A number of original articles also describe the use of mesoporous silica NPs in the role of delivering anti-cancer drugs doxorubicin, abciximab and epirubicin [323,324,325]. It is noteworthy that the last article involved the authors conducting research on combinations of antitumor and antibacterial drugs with mesoporous silica NPs. This study is of particular significance in light of the heightened immune suppression and resistance to antibacterial pharmaceuticals observed in cancer patients, rendering them more vulnerable to bacterial infections of various kinds [326]. The team of authors demonstrated increased antibacterial activity, as evidenced by the synergistic effect of these drugs on different bacterial models. It was noted that the most effective result was achieved for NPs functionalized with epirubicin and ofloxacin [325].

### 7.5. Carbon Particles and Quantum Dots in Pharmacy

As previously referenced, fullerenes initiated a trend for their application in immunology in the mid-1990s [110]. This trend has continued to develop in modern scientific researches, suggesting the application of fullerenes as adjuvants for vaccines. Scientific groups have demonstrated the implementation of C_60_ fullerenes as adjuvants enhances the immunogenic properties of immunological pharmaceuticals, as evidenced by studies on various vaccines against viral infections, including the COVID-19 and hepatitis C virus [327,328]. Furthermore, the anti-inflammatory effect of C_60_ fullerenes on mice models of atopic dermatitis has been described by Shershakova et al. [329]. It is hypothesized by scientists that the use of fullerene aqueous dispersions leads to the restoration of skin barrier function [329]. The targeted delivery of APIs is an area of active investigation, based on the physicochemical properties of fullerenes, namely their size characteristics and specific surface area. The efficiency of using different fullerene modifications as a delivery system for drugs of different pharmacological groups is described in a number of papers [113,330,331].

Quantum dots of different origins are applicated in studies of targeted drug delivery due to their small size (less than 10 nm), variability of modifications and large specific surface area, which renders them suitable for application in pharmacy. Thus, the review article describes a wide range of applications of quantum dots in medicine and pharmacy, pointing out the possibility of their use not only in drug delivery, but also as biosensors for various biomolecules and assistants in anti-cancer therapy [332]. Turning to original articles, the application of quantum dots as an effective delivery system for the anti-cancer drug methotrexate is considered. It is known that cancer cells are able to develop resistance to various pharmaceuticals, which complicates the course of treatment [333]. The paper describes the enhanced efficacy of methotrexate conjugated with CdSe quantum dots to reduce the resistance of cancer cells to chemotherapeutic drug [334]. The scientists have determined that the minute size of the quantum dots activates the endocytic transport pathway, thereby enhancing the penetration of the chemotherapeutic agent into target cells [334]. In the context of drug resistance, quantum dots have also been employed against bacterial infections, exhibiting noteworthy antibacterial properties [335,336].

Important application of quantum dots in pharmacy is their use in the quality control of drugs. Liquid chromatography frequently necessitates the use of harmful organic components, which is incongruent with the current trend of green chemistry. However, in the following work, researchers have successfully introduced carbon quantum dots into a water-based liquid chromatography method [337]. Depositing hydrophilic quantum dots synthesized with different functional groups on silica gel, the researchers were able to achieve successful separation of compounds with different polarity. Due to their unique luminescent properties, quantum dots have found application in spectrofluorometric analysis, as demonstrated by the example of the antimicrobial drug nifuroxazide, where the suppression of luminescence of quantum dots upon addition of drug made the pharmaceutical analysis possible [338]. Furthermore, a colorimetric platform for the detection of captopril substance was created using molybdenum quantum dots [339].

### 7.6. Applications of Peptide Modified NPs

From a pharmaceutical perspective, the most common application of NPs modified with peptides is in the field of anticancer therapy, specifically targeting drug delivery. Several articles describe the advantage of reduced toxicity and selective drug delivery to tumor tissues [340,341,342]. A group of such peptide molecules has been named “cell penetrating peptides” (CPP) [343]. A study presents a model of peptide modified NPs in complex with encapsulated doxorubicin [344]. The authors demonstrated that this collaboration enhances intracellular and intranuclear delivery of API to cancer cells. This property of CPP is extremely important if the therapy is aimed against cells with high drug resistance [344].

The development of vaccine-type immunobiological pharmaceuticals necessitates the fine-tuning of the final substance to minimize undesirable reactions. In this regard, peptide in complex with NPs have emerged as a promising solution due to their inherent chemical modification capabilities. The latest articles devoted to the search for a safe vaccine against fever caused by the Dengue virus describe both successful clinical trials of a vaccine based on complex of AuNPs and synthetic peptide [345] and the prospects for the development of peptide modified NPs in the field of immunology to create new safe vaccines [346]. A scientific work describes the production of a mesoporous silica NPs modified with peptides containing epitopes of the SARS-CoV-2 virus [347]. The results demonstrated effective delivery of epitope peptides by mesoporous silica NPs, as well as a strong humoral and cellular immune response during preclinical trials [347].

Another application of NPs modified with peptides in pharmacy pertains to their utilization as antimicrobials, a field of increasing importance due to the proliferation of multidrug-resistant pathogens, a major challenge for the global pharmaceutical industry. The solution to this problem is proposed in [348,349]. The review article discusses the positive aspects of using antimicrobial peptides in conjunction with inorganic NPs [348]. The authors of the research conducted several laboratory studies of a new nanosystem based on a complex of peptides with antimicrobial activity and dextran NPs, which was aimed against *Pseudomonas aeruginosa* [349]. The results of these studies demonstrated high pharmacokinetic characteristics, indicating the effectiveness of the proposed peptide modified NPs [349].

## 8. Quality Control of NPs as Pharmaceuticals

It is important to understand that pharmaceutical chemistry solves several key tasks, from the design of drugs to the selection of methods for their synthesis, but the most important of them is the quality control of pharmaceutical substances. Since NPs are actively applied in the pharmaceutical industry and offer a wide range of possibilities for producing modern drugs, they have all the same requirements as conventional drugs: activity, selectivity, purity, stability and low toxicity. Consequently, in a similar way to pharmaceuticals, nano-drugs must be subjected to strict quality control by means of various physicochemical methods.

### 8.1. Evaluation of the Morphological and Topographic Characteristics of NPs

Following the synthesis of NPs, it is necessary to verify their morphological properties, such as size, shape, and surface area. In the field of pharmaceutical analysis, several methods are used to control these parameters and each of them will be considered in the present section.

#### 8.1.1. Electron Microscopy

**Figure 18:**
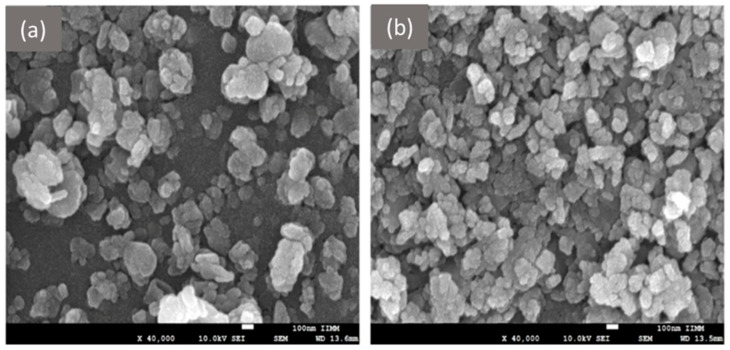
An example of images ZnO NPs obtained with SEM. A detailed interpretation of the figure is presented in the article [351].

Electron microscopy (EM) techniques are used for visualizing NPs. Scanning electron microscopy (SEM) based on the emission of X-rays and electrons from the surface of the object, as induced by the application of an electron beam to the sample, facilitates the acquisition of high-quality three-dimensional images of NPs samples [350]. Figure 18 demonstrates an example of visualization of ZnO NPs using SEM.

**Figure 19:**
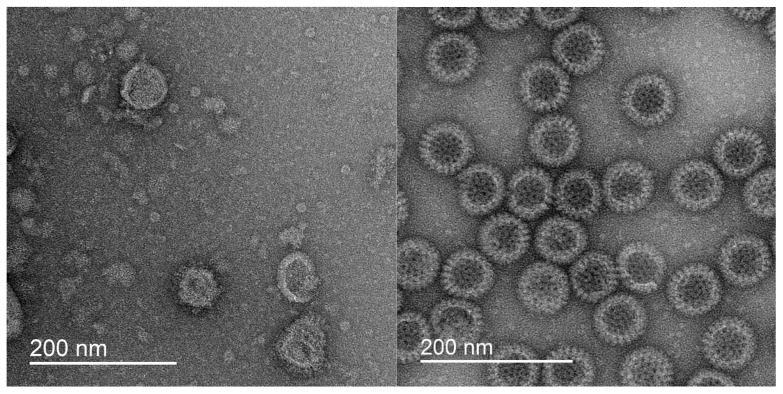
An example of VLP images obtained with TEM. A detailed interpretation of the figure is presented in the article [126].

Transmission electron microscopy (TEM) is a technique that provides a two-dimensional image of the NPs. The principle of transmission microscopy is to focus an ultra-thin electron beam on an ultra-thin section of an NP sample. The beam’s interaction with the sample causes some of the electrons to be scattered and some to penetrate the sample and fall on the microscope’s fluorescent screen [352]. Figure 19 demonstrates an example of visualization of VLP using TEM.

**Figure 20:**
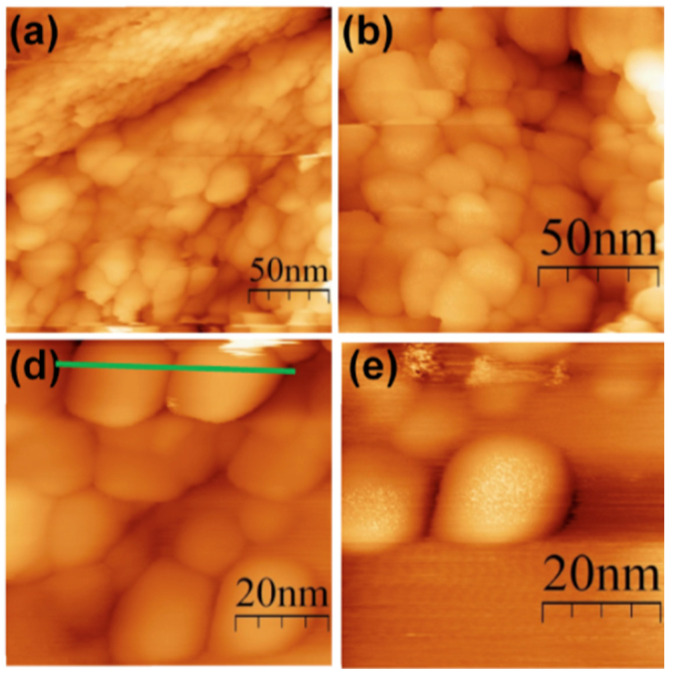
An example of AuNPs images obtained with STM. A detailed interpretation of the figure is presented in the article [355].

Scanning tunnelling microscopy is based on the phenomenon of quantum tunneling. Voltage is applied through the metal tip, which is closely connected to the surface of the NP. This process involves the extraction of electrons from the surface of the sample. The generation of an electric current is used for surface visualization, making it possible to evaluate the topographical characteristics of the NPs [353]. This type of EM is most commonly used to visualize inorganic particles, but it causes difficulties in scanning particles of biological origin [354]. The visualization of AuNPs is demonstrated on Figure 20.

#### 8.1.2. Dynamic Light Scattering

Dynamic Light Scattering (DLS) is the most common method employed for the control of particle size characteristics. The study is conducted on solutions that contain a dispersed particle phase. This method is based on photon correlation spectroscopy, based on the analysis of the Brownian motion of particles in a dispersed medium. This leads to fluctuations in the local concentration of particles, inhomogeneities of the refractive index and the intensity of scattered light [177]. The particle size is calculated using the Stokes-Einstein equation:

(8) $$D=\frac{k_{B\;}T}{6\pi \eta r}$$

where *D* is the diffusion coefficient, $k_{B}$ is the Boltzmann constant, *T* is the absolute temperature, η is the viscosity, and r is the particle radius.

Depending on the capability of the analysis equipment, this method allows detecting the size of nanostructures in the range from 1 nm to 10 μm [180,356]. A significant number of research groups have adopted the DLS analysis to obtain the dimensional characteristics of the synthesized NPs. The work of a group of researchers focused on obtaining and controlling polymer NPs coated with hyaluronic acid, DLS analysis acted as a method for controlling the dimensional characteristics of these particle [357]. The potential for determining the qualitative characteristics of nano-pharmaceuticals using DLS analysis has been described in the next article.

An important criterion for the storage of immunobiological pharmaceuticals is the temperature range of +2 to +8 °C. Deviations from these temperature ranges can result in the coagulation of protein particles and their subsequent aggregation. The utilization of DLS method to regulate alterations in the dimensional characteristics of nano-drugs with divergent storage modalities appeared to be a rational approach. In scientific article [177], the results of a comparison were demonstrated between the dimensional characteristics of interferon α2-b of appropriate quality and the interferon subjected to coagulation by heating to 100 °C for 5 min. The outcome of this analysis revealed a substantial increase in the particle size of the coagulated interferon, in comparison to the native sample.

#### 8.1.3. ζ-Potential

As previously outlined, a significant characteristic of NPs is their surface charge, which allows to make conclusions about possible interactions between particles in colloidal solutions. The electrical potential at the EDL boundary, otherwise known as the Zeta Potential, allows to assess the interaction of particle with biomolecules, as well as their stability, by characterizing the electrostatic repulsive forces between NPs. Particles exhibiting a high electric potential value of more than +30 mV or a low electric potential value of less than -30 mV demonstrate strong cationic and anionic properties, respectively, thereby indicating the stability of nanostructures [358]. This knowledge of the properties of NPs can be applied to the prediction of targeted drug delivery. The review paper emphasizes the necessity to investigate the surface electrical properties of particles, which can change during the adsorption of surfactants and APIs, for their effective application as drug carriers [359]. In their study, the authors of the article resorted to a stepwise measurement of the zeta potential immediately after synthesis, 24 h later, and 5 days later to investigate the stability of the synthesized solid lipid NPs [360]. The researchers obtained a value of |±30 mV| and selected the most suitable components for synthesis.

#### 8.1.4. The Brunauer-Emmett-Teller (BET) and Barrett-Joyner-Halenda (BJH) Methods

**Figure 21:**
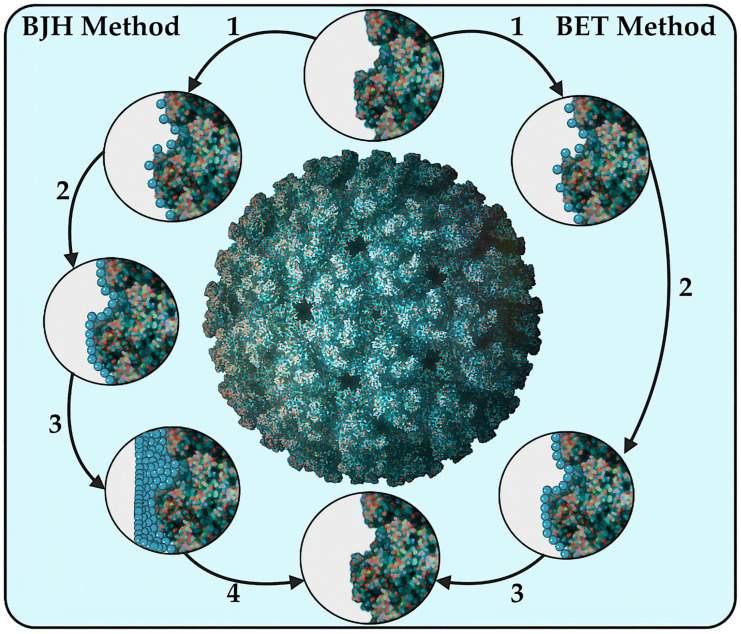
Graphical representation of the principle of operation of BET and BJH methods, where ‘1’ is the adsorption of nitrogen on the surface of the NP; ‘2’ is the formation of a monolayer; ‘3’ is the desorption of nitrogen from the surface of the NP in BET method, or complete filling of all NPs pores with nitrogen in BJH method; ‘4’ is the desorption of nitrogen from the surface of the NP in BJH method.

To characterize the topological properties of NPs, two known methods based on the processes of adsorption and desorption of liquid nitrogen are applied. The BET method is utilized to estimate the porosity of particles and their surface area (Figure 21). The particles are cooled with liquid nitrogen, and a monolayer is formed as a result of the adsorption process. When the particles are heated, the nitrogen is released, and desorption occurs. The release of nitrogen is therefore the quantitative indicator that allows calculation of the surface area of the sample [361].

A more modified method for a detailed investigation of the surface of NPs, based on Brunauer’s idea, was proposed in 1951 by the scientists E. Barrett, L. Joyner and P. Halenda [362]. The BJH method involves prolonging the interaction between nitrogen and the NP surface, leading to the condensation of nitrogen molecules within the NP pores under increasing pressure (Figure 21). Upon reaching the saturation point, the pores of the sample are filled with liquid nitrogen molecules. According to a similar BET technique, desorption occurs upon heating, and according to the obtained indicators, it is possible to obtain the dimensional characteristics of the pores using the Kelvin equation [361].

### 8.2. Control of the Chemical Structure of NPs

A structural analysis of the obtained NMs is necessary to control the final product. It is imperative to comprehend the process, how the aggregation of molecules in the NPs passed, whether the substances adsorbed on the surface were correctly selected, which functional groups were present on the particles, and whether impurities were present in the synthesized samples. In the field of pharmaceutical chemistry, spectroscopy and X-ray fluorescence analysis are used to carry out the tasks described above.

#### 8.2.1. IR-Spectroscopy

The analysis method based on the infrared region of the spectrum has the potential to characterize NPs of various origins, from inorganic particles to NPs of biological origin. IR spectroscopy is most often associated with the analysis of chemical structure through functional groups, however, when applied to the nanoscale, this method also allows the determination of particle size characteristics [363]. The implementation of IR analysis for the purpose of quality control of NPs can be achieved through the application of a singular technique or a comprehensive array of techniques. The canonical purpose of IR spectroscopy is the identification of functional groups, which allows the analysis of the chemical structure of substances fixed on the surface of NPs. This approach is important for tracking changes in the modification of nano-pharmaceuticals. For example, a scientific study [364] describes the use of IR spectroscopy to determine temperature-dependent structural changes in AuNPs coated with D-penicillamine. A significant number of studies have been conducted with the objective of investigating the potential of the application of IR analysis to provide information of NPs structural features. The authors of the article demonstrate the application of near-infrared spectroscopy with Fourier transform in combination with the classification of samples based on spectral features using the inverse error propagation of an artificial neural network to estimate the size of TiO_2_ particles [363,365]. Furthermore, differences in the IR spectra can serve to assess the stability of the obtained nano-pharmaceuticals, acting as markers of degradation or other changes in the properties of NPs [366]. Despite the common perception of IR spectroscopy as a qualitative analytical method, contemporary approaches to its application enable the acquisition of quantitative data. For example, it can be applicated for the concentration assessment of functional groups or molecules associated with NPs [367].

#### 8.2.2. Raman Spectroscopy

Another spectroscopic method for analyzing the chemical structure of NPs is Raman spectroscopy. This method is based on the study of photon and phonon oscillations during monochromatic laser irradiation of the sample surface [368]. The data obtained from this process can be used to make statements about the chemical and intermolecular bonds of the investigated objects. A publication shows the possibility of separating carbon NMs of different sizes by combining Raman spectroscopy and capillary electrophoresis [369]. The scientific publication describes an integrated approach to the control of synthesized Fe_3_O_4_ NPs [370]. The authors obtained a Raman spectrum with characteristic NPs peaks.

In the majority of original works, NPs are applied as substrates for the determination of various pharmaceutical substances using Raman spectroscopy. In a case of the determination of fentanyl derivatives, the authors used nanocrystals containing Au and Ag [371]. The modification of Raman spectroscopy with an improved surface allows a more sensitive and selective analysis of the molecular structure [368]. A biosensor for the detection of C-reactive protein, a biomarker of inflammatory reactions, was constructed based on a combination of this modification with a substrate consisting of AuNPs [372].

#### 8.2.3. X-Ray Fluorescence Analysis

X-ray fluorescence analysis is an effective method of assessing the chemical composition of the synthesized particles. The method is based on irradiating the sample under study with X-rays, which excites the atoms of the substance and leads to the transition of electrons from the lower orbitals to the upper ones. This process results in the formation of a vacant orbital, which is subsequently filled with electrons from the outer shells. This leads to the release of energy, followed by the emission of photons [373]. The application of X-ray fluorescence analysis to characterize the elemental composition of AgNPs and TiO_2_ NPs was demonstrated [374,375]. In the mentioned study the synthesized mesoporous silica was used as a support for CuO and AgNPs after calcination. The X-ray fluorescence indicated the presence of CuO and AgNPs species at different contents. The initial composition of each sample was shown to control the particle size, with the Ag–Cu-Mesoporous silica content ratio [374]. The X-ray fluorescence method was applied to investigate the elemental/chemical composition of the finished product, which indicates the presence of Ti and Cu in the synthesized Cu-doped TiO_2_ NPs [375].

The other example demonstrates the composition of bioactive glass and biphasic calcium phosphate nanopowders mixtures which was analyzed applying X-ray fluorescence analysis [376]. X-ray fluorescent analysis showed that the prepared bioactive glass had as-predicted composition equal to 36.9 SiO_2_, 57.6 CaO, and 4.24 P_2_O_5_ (wt%). X-ray fluorescence computed tomography, as one of the varieties of X-ray fluorescence spectroscopy, makes it possible to identify, quantify and localize elements inside nanoobjects by detecting X-ray fluorescence. The study demonstrating the use of desktop X-ray tomography to accurately image the distribution of AuNPs injected into a tumor-bearing mouse [377]. An interesting example is the Monte Carlo modeling results will provide a guide for developing an optimal benchtop X-ray fluorescence imaging system for in vivo preclinical imaging, depending on the sizes of AuNPs, their concentrations, and radiation doses [378].

### 8.3. Evaluation of the Optical Properties of NPs

#### 8.3.1. UV-Spectroscopy

The most common methods for assessing the optical properties of NPs are ultraviolet (UV) spectroscopy and UV-visible spectroscopy. The principle of the methods is based on the absorption of UV radiation by molecules in the NPs, and the absorption of light itself occurs due to the transition of electrons from the ground state to the excited state. The classification of particles reveals a wide range of characteristics, including their nature, origin, shape, and size, which collectively serve as indicators of their distinct optical properties. The unique properties of surface plasmon resonance render UV spectroscopy the simplest and most effective method of controlling the quality of inorganic NPs. The UV spectrum of the synthesized CuO particles was the subject of study in [379]. The study revealed an absorption peak at 340 nm, which is characteristic of the compound and serves as confirmation of the formation of NPs. The study of the release of a hypoglycemic pharmaceutical from polymer particles was conducted using UV spectroscopy in work [380]. The Bradford colorimetric method, a classic technique closely related to the application of UV spectroscopy, is employed to determine the concentration of protein particles in a solution. This method was applied by a research group that was working on the synthesis of protein NPs from bovine serum albumin [381]. The authors acknowledge the efficacy of this method in determining the concentration of protein particles. However, they emphasize the necessity for comparison with other physicochemical methods, as the accuracy of the results may be compromised.

#### 8.3.2. Fluorimetry

The fluorimetry method has also been applied to evaluate the quality of NPs. The method is based on the excitation of a sample by electromagnetic emission, and the measurement of the intensity of light emitted by the sample in the UV-visible or near-IR regions of the spectrum. The luminescence phenomenon is initiated by the transition of electrons from an excited energy level to the main one, accompanied by energy dissipation in the form of electromagnetic emission. The absorption maximum determined by UV spectroscopy is utilized in fluorimetric measurements to select the excitation wavelength. Fluorimetry finds application in the development and utilization of quantum dots used for the diagnosis and treatment of tumors [382,383]. Furthermore, the method is indispensable in the quality control of drugs based on such NPs [384] and in the final visualization of fluorescence of edge points targeted in tumor tissues [385,386]. The fluorimetry method has been demonstrated to be able to detect the inclusion reaction of pharmaceutical molecules in the delivery system, as exemplified by the formation of the inclusion compounds mangiferin-humic complex and favipiravir-humic complex [387]. This reaction was accompanied by quenching of fluorescence and second-order scattering of the initial humic complex, in proportion to the concentration of the guest compound. It is noteworthy that the phenomenon of fluorescence quenching can also quantify the interaction of macromolecules, as evidenced by the binding of fullerenol to alcohol dehydrogenase and human serum albumin [388].

### 8.4. Evaluation of the Thermal Properties of NPs

#### 8.4.1. Differential Scanning Calorimetry

Differential scanning calorimetry (DSC) is a highly efficient analytical method that is widely used in pharmaceutical chemistry to study the thermodynamic properties of pharmaceutical substances. The method is based on measuring the difference in heat fluxes between the test sample and the reference one depending on the temperature change [389]. The primary field of application of DSC for the analysis of NPs are the study of thermal stability, the analysis of phase transitions, the evaluation of the effect of particles on the thermal properties of drugs, the study of polymeric drug delivery systems. For example, a DSC study of the distribution of indomethacin, which is a part of lipid NPs, was shown in [390]. Another application of DSC is demonstrated in scientific work, which shows the possibility of analyzing changes in the crystalline state of azithromycin obtained in the form of nanosuspensions [391]. The DSC method was also used to demonstrate the preservation of the crystal structure of dapsone nanocrystals and their thermal stability, which renders these particles suitable for use in new dosage forms. The DSC results also confirmed the assumption that the process of obtaining nanocrystals does not lead to significant changes in the physicochemical properties of pharmaceuticals, which is important for ensuring therapeutic efficacy [392].

#### 8.4.2. Thermogravimetry

Another variation of thermal analysis is thermogravimetry, which enables the investigation of the thermal stability of NPs and NMs. This method is based on measuring the change in mass as a function of temperature [393]. This approach enables the characterization of the thermal stability of synthesized NMs [394,395]. In a study of the immunosuppressor complex with mesoporous silicon particles, the authors performed a thermogravimetry to assess its thermal stability [396]. The outcome of this analysis was a comparison of the masses of NPs in the absence and presence of pharmaceutical, with the discrepancy between these masses serving as an indicator of successful drug encapsulation.

### 8.5. Intrinsic Radiothermal Emission of NPs

The unique property of NPs and NMs to emit in the millimeter wavelength range, the physics of which has been described above, has been proposed as an approach for the quality control of pharmaceuticals consisting of biologically active NPs [126]. This method is based on the detection of the particles’ intrinsic radiothermal emission. The data obtained make it possible to evaluate not only the qualitative, but also the quantitative properties of nano-pharmaceuticals [177]. Humic–fulvic acids have demonstrated efficacy as therapeutic and preventive nutrition, exhibiting antiviral, immunomodulatory, and detoxifying properties [397,398]. Since humic acids are most often realized in the form of nanodispersions, with the actual size of these nanocomplexes reaching 400 nm [398], the authors of the article proposed an approach to evaluate the qualitative characteristics of industrial variations of therapeutic nutrition with humic–fulvic acids based on their intrinsic radiothermal emission [180]. The study revealed that the density of the emission flux varied between samples with proper shelf life and with expired shelf life. The article demonstrates the application of a method for the investigation of intrinsic radiothermal emission, employing a case study of immunobiological pharmaceuticals comprising various types of interferons and VLP vaccines [177].

## 9. Discussion and Conclusions

The discovery of the nanoscale was perhaps a defining moment for science. The development of nanotechnology, which began in the middle of the last century, continues to this day, allowing scientists to solve non-trivial tasks in their industries. The application of ultra-small particles is indeed widely known, ranging from technical to medical and pharmaceutical fields of science [399]. However, for the purposes of this review, it is proposed to focus on a narrower discussion of the use of NPs, specifically in the pharmaceutical industry.

Most articles selected for this review demonstrate the possibility of using NPs of different origin for drug delivery [59,62,272]. This application became possible due to the unique physicochemical properties of the particles and the possibility of flexible design with subsequent modifications [224], which were described earlier in the review. The delivery of chemotherapeutic drugs in the treatment of oncological diseases is of the greatest interest. For such classes of drugs, several requirements are proposed, including increased pharmacokinetic parameters, improved pharmacodynamic properties, and reduced side effects [55]. Consequently, LNPs, NPs modified with peptides, and polymer NPs emerge as optimal candidates, exhibiting not only high biocompatibility but also enhanced intracellular delivery and controlled release [55,297,343]. The flexibility of their design, which consists in the possibility of adding specific ligands to the surface of NPs [253,299] or constructing peptide chains with specified characteristics [278], is also a weighty argument when choosing them as drug carriers. However, it is important to note that despite the importance of the research and development of modern anticancer agents based on NPs and NMs, the literature review presented shows that the aspect of drug delivery by ultra-small particles is quite broad and affects their combined use with drugs of different pharmacological groups [113,293,301,318]. For instance, the present review discussed the application of AgNPs in conjunction with antibacterial agents as a strategy to address the global challenge of antimicrobial resistance in microorganisms [318]. Due to the properties of increased loading capacity, fullerenes are actively used for carrying various pharmaceuticals to increase their therapeutic activity [29]. Along with LNPs active application in anti-cancer therapy, they are also used in the creation of immunobiological drugs for effective mRNA transport [400]. And combinations of lipid and polymer particles are utilized to solve non-trivial tasks for the delivery of protein pharmaceuticals degraded in the gastrointestinal tract [301].

As demonstrated above, there is a broad range of ways in which various types of NPs can be applied in different therapeutic contexts. It is important to note, however, that each type of NPs have its own advantages and disadvantages. It is notable that LNPs, polymer NPs and VLPs exhibit high biocompatibility and biodegradability [70,400,401]. However, as indicated by the authors of several articles, there are also limitations and disadvantages to consider [402,403]. The immunogenicity of artificial NPs is a well-documented phenomenon, signifying their capacity to stimulate the immune response of the host to foreign agents [404]. In order to overcome this limitation, a variety of approaches are used to produce biomimetic NPs by modifying their surfaces [405]. However, it is also important to consider the influence of various phenotypic signs on the immune response of the host to injected nanopharmaceuticals [404].

A significant challenge to the application of NP-based pharmaceuticals in clinical trials is the limited knowledge surrounding their long-term toxicity, particularly in view of the predominance of short-term studies in the published literature [402]. Indeed, the absence of research on chronic toxicity can result in significant consequences in the future, underscoring the necessity for heightened attention to this subject in subsequent studies. The toxicity of inorganic particles, such as AgNPs and quantum dots, is frequently referenced in the extant literature [406,407]. A number of articles offer suggestions for resolving these issues, including the adoption of green chemistry methodologies for synthesizing the inorganic particles [408,409], and, in the case of quantum dots, the introduction of protective inorganic surface layers to reduce their cellular toxicity [407].

The primary factors contributing to the heightened toxicity of particles are their capacity to accumulate in organs and tissues, and the ability of certain NPs to penetrate various biological barriers [410]. Conversely, the accumulation of particles within the body is also regarded as their advantage, given that the primary objective of targeted delivery is to direct them towards unhealthy tissues. However, it should be noted that a number of physical and chemical factors may result in deviations from the set target. These factors include, for example, the size of the particles, their shape, surface charge and its modifications. Accumulation in healthy tissues and organs (liver, kidneys, spleen, lungs and, in some cases, the brain) may occur as a result [411]. Despite the effectiveness of some NPs, the possibility of spontaneous release of the active substance should not be excluded, which may render it difficult to obtain reliable results during clinical trials [401].

However, the limitations affect not only the medical aspect of NPs applications, but also the pharmaceutical field. To illustrate this point, consider the issue of scalability in the context of manufacturing processes, from the laboratory to the factory [402]. The primary challenges pertain to reproducibility, quality control, cost implications, and technological constraints. It is imperative that NPs properties are monodisperse, given the established correlation between their biodistribution, efficiency and toxicity and their physico-chemical properties. For instance, LNPs employed in mRNA vaccines necessitate precise size control, ranging from 80 to 100 nm [402]. The process of transferring techniques for modifying known NPs from laboratory to industrial conditions is often fraught with difficulties [412]. The capacity to incorporate an active pharmaceutical ingredient into particles is unquestionably advantageous. Nevertheless, from a pharmaceutical chemistry perspective, there are several constraints associated with the solubility of the drug and the composition of the NP matrix [410].

As previously mentioned, the unique physicochemical properties of NPs should be discussed in more detail. The morphological and topographic characteristics of the particles evidently play a major role in understanding their future interaction with organism cells. Indeed, the capacity of these particles to penetrate diverse biological barriers is contingent on their size [123]. The topography of particles is also an important characteristic, which is directly related to the SSA, describing the further efficiency of pharmaceuticals transport [140]. In addition, different particle form factors allow to extend their capabilities in the field of drug delivery and their application as independent pharmaceutical agents, varying their pharmacokinetic and pharmacodynamic properties and improving their accumulation in specific organs [128].

In view of the potential accumulation, it seems worthwhile to take a more responsible approach to the process of NPs synthesis and avoid their increased toxicity, to comply with the well-known principle of medical ethics “Noli nocere”. In the synthesis of ultra-small particles, it is necessary to consider their interactions between themselves in order to understand their possible aggregation and to assess their stability [159]. The extant types of intermolecular interactions of NPs were discussed in detail in Section 4.4. In addition to the fundamental Van der Waals forces, which can be employed to describe the behavior of particles in dispersed solutions, there are equally fundamental theories of DLVO, JKR and DMT. Theories based on Van der Waals interactions, provide additional insights into the behavior of particles, complementing their connection with EDL and mechanical properties [157]. However, these theories have a number of limitations (see Section 4.5.1, Section 4.5.2 and Section 4.5.3), which makes it impossible to apply only one of them, forcing scientists to consider three options in parallel [166]. Despite their limitations, they are important tools for studying the stability of NPs and their ability to aggregate, which is necessary to produce particles for pharmaceutical applications.

One of the final physical properties of biologically active NPs and NMs to be described is their radiothermal emission [126]. This property is based on the formation of quasi-plasma regions on the surface of NPs, which, like plasma irradiation, will be emission centers and will emit in the millimeter range [177]. This irradiation will be an individual characteristic of the sample, which will allow the use of this property as a method of quality control.

The synthesis of NPs and NMs merits particular consideration, as the assessment of their quality will be contingent on the nature of the origin of the samples. In this review, the most commonly used approaches for the production of particles for pharmaceutical applications have been discussed. The concept of “design flexibility” was mentioned, which refers to the fine tuning of the final product based on the functions assigned to it [224]. Microfluidic methods allow to obtain particles of a strictly defined size [196], methods of peptide synthesis, as future modifiers for NPs, from solid phase to recombinant allow to select the necessary amino acid residues [278,281], and nanoprecipitation allows to vary the synthesis conditions to obtain polymeric NPs with the required properties [207]. Despite all the complex and not very sophisticated methods of particle synthesis, it is worth considering the environmental consequences. The process of producing NPs involves the use of various organic compounds and their derivatives, as well as hazardous chemicals, which leads to environmental pollution [413]. However, recent advancements have enabled the use of environmentally friendly materials that do not require special disposal. The synthesis of NPs can be achieved through the application of microorganisms [414], plant extracts [415], etc. Green chemistry, a contemporary approach to synthesizing numerous pharmaceutical substances. However, it is yet to encompass all possibilities of organic synthesis and needs to be further developed.

The quality control methods discussed in the review are indispensable for the assessment of the qualitative and quantitative characteristics of NPs synthesized for pharmaceutical purposes. The modern hardware equipment in leading pharmaceutical laboratories has rendered it possible to assess all the necessary physicochemical characteristics of particles [350,357,363,380]. In addition to the instrumental methods of physical and chemical analysis, it is also imperative to consider the toxicological aspect. Despite the increased biocompatibility of the particles, it is worth considering the possible side effects and perform a toxicological analysis too [411,416,417]. A direct discussion of the instrumental methods of analyzing the qualitative and quantitative characteristics of NPs reveals two main issues. Firstly, the complexity of the methods, and secondly, the necessity of long sample preparation times. Furthermore, it is evident that the vast majority of the aforementioned methods do not permit non-invasive analysis, a task that seems to be unattainable for the monitoring of manufactured drugs based on NPs. The last of the considered quality control method, which is based on the detection of its intrinsic radiothermal emission, unlike the others, allows noninvasive assessment of drug quality characteristics without opening the primary packaging [177]. This method is a relatively recent approach for pharmaceutical characterization, so it is worth considering its specificity, which is directed strictly at biologically active NPs of a complex shape, as well as the need for calibration for a specific sample and the complementation of results from other physicochemical highly validated methods.

Despite the considerable increase in popularity of NPs and NMs in medicine and pharmacy, it is important to emphasize the necessity of their appropriate regulation. A review of the scientific literature reveals that there is considerable variation in the reported size ranges of various NPs [418]. While such disparities in definition may appear inconsequential to some scientists, it is imperative to recognize the critical importance of establishing uniform international standards for the subsequent proper use of nanopharmaceuticals as finished medicinal products [419].

From the standpoint of the pharmaceutical industry, there is a necessity for the validation of methodologies employed in the evaluation and testing of NPs. It is important to note that existing methods are not always able to adequately assess the toxicity or qualitative characteristics of the final materials [420,421]. Also, it has been demonstrated that preclinical trials do not always consider the complexity of the interaction of nanopharmaceuticals with various human organ systems, as well as interference with reagents used in vitro [422]. The absence of a robust preclinical foundation can result in deleterious consequences during clinical trials.

Another factor that requires proper regulation is the impact of nanomedical drugs on the environment [419]. It is evident that a multitude of consequences arise from the impact of pharmaceuticals on the environment [423,424,425]. It is erroneous to equate the circulation of nanopharmaceuticals in the environment with that of their non-nano analogues without undertaking a comprehensive study of the issue [426]. It follows that a comprehensive understanding of the environmental impact of such pharmaceuticals is imperative.

At this stage, most countries, including the United States and the European Union, are issuing guidelines to regulate the production of pharmaceutical products containing nanostructures. For example, in 2022, the FDA updated the industry guidelines, which address the basic requirements attributed to nanopharmaceuticals [427]. The European Union also issues various recommendations for the definition of NMs and guidelines for their production [428]. The European Medicines Organization is actively promoting the regulation of nanopharmaceuticals by developing several recommendations for the production of nanomedicines, as well as forming a group of Nanomedicine Experts responsible for developing standards for the evaluation and registration of such products [419]. In contrast, other countries have adopted a more uniform legislative approach for the regulation of pharmaceuticals based on NPs [421]. The advent of nanotechnology within the realms of medicine and pharmacy has precipitated a rapid transformation in the therapeutic treatment of numerous pathologies. However, the optimal utilization of this technology is contingent upon the establishment of a harmonized international regulatory framework and collaborative endeavors between research communities and regulatory authorities. This approach will facilitate the production of high-quality, safe and effective nanopharmaceuticals.

The modern pharmaceutical market is already implementing a number of drugs containing various classes of NPs. In the absence of an official categorization of the pharmaceutical market according to the specific field of application, it is important to examine the current state of the industry. For example, in one of the review work, there are five main segments: delivery of medicines, therapeutic means, diagnostics in vitro, visualization in vivo and implants [429]. At the same time, in the next review article, the authors indicate an additional segment of “active implantation” [430]. In both cases, the majority of the market share for nanodrugs (more than 30%) is accounted for the delivery of medicines. The combination of such market indicators with the rapidly developing scientific interest in targeted drug delivery may signal the relevance of this segment. However, as previously outlined, a harmonized categorization of such pharmaceuticals based on their field of application necessitates the establishment of consolidated regulatory documentation.

According to the ClinicalTrials database, approximately 200 clinical trials related to the testing of pharmaceuticals based on NPs are currently ongoing at various stages of completion. Notwithstanding the aforementioned clinical trials, a number of drugs based on NPs have already been approved by the FDA. One of the earliest nanodrug to be approved by the FDA was Gris-PEG^®^, an antifungal drug based on a nanocrystal, in 1975. As a consequence, the aforementioned drug has become the starting point for the development of a significant number of other drugs based on various NPs. For example, CosmoFer INFeD Ferrisat^®^ represent the first metal-based nanopharmaceutical of iron dextran colloid, Doxil^®^ the first antitumor liposomal nanodrug, which were developed in 1995. Furthermore, Vyxeos^®^, an antitumor drug based on daunorubicin, and BNT162b2 and mRNA-1273, two liposomal nanovaccines, which were developed in 2021 [431].

To conclude this review, it is important to note the considerable increase in interest among researchers in the field of pharmacy in the area of nanotechnology. A meticulous analysis of the publications pertaining to NPs applied in pharmacy, based on PubMed data, reveals a trend towards a significant increase in researchers’ interest. For example, at the beginning of the 21st century, publications on “nanoparticles as drugs” were just beginning to gain popularity (500 articles in 2005), and every year the number of scientific papers only increases (8000 articles in 2024). The contemporary pharmaceutical industry is a dynamic and constantly evolving field, exploring new approaches to the treatment of a wide range of diseases. In this context, NPs emerge as a universal tool to improve therapeutic outcomes by controlling pharmacodynamic parameters and reducing side effects due to the increased biocompatibility of certain types of particles. A comprehensive review of the extant literature has revealed the contemporary prospects for the advancement of pharmacy, immunobiology and, indeed, the field of medicine. It is noteworthy that several of the nano-pharmaceuticals described in this review are already undergoing clinical trials, indicating a positive trend in the development of nanopharmaceuticals in the future.

## 10. Future Directions

It is evident that even at the beginning of the last century, it was challenging to conceptualize the prospect of regulating the trajectory of pharmaceuticals within the human organism, and now it is practically possible. However, the pharmaceutical use of NPs has not been fully investigated and not all possible modifications to deliver the APIs directly to the target organs have been created. A positive prediction can be made about the further development of nanopharmaceuticals as a new field of science, or nano-drugs as a new generation of pharmaceutical substances, based on the research carried out in the available scientific literature. To further develop this area, there is a necessity to improve and find new ways to synthesize NPs and NMs. The scientific studies presented in the paper addressed comprehensive approaches to the characterization of the produced particles. Only this approach will allow the development of new drugs with improved properties.

## Data Availability

No new data were created or analyzed in this study. Data sharing is not applicable to this article.
